# Process Intensification Strategies for Esterification: Kinetic Modeling, Reactor Design, and Sustainable Applications

**DOI:** 10.3390/ijms26157214

**Published:** 2025-07-25

**Authors:** Kim Leonie Hoff, Matthias Eisenacher

**Affiliations:** Circular Transformation Lab, Technische Hochschule Köln, Campus Leverkusen, 51379 Leverkusen, Germany

**Keywords:** heterogeneous catalysis, pervaporation, membrane reactor, process intensification, esterification

## Abstract

Esterification is a key transformation in the production of lubricants, pharmaceuticals, and fine chemicals. Conventional processes employing homogeneous acid catalysts suffer from limitations such as corrosive byproducts, energy-intensive separation, and poor catalyst reusability. This review provides a comprehensive overview of heterogeneous catalytic systems, including ion exchange resins, zeolites, metal oxides, mesoporous materials, and others, for improved ester synthesis. Recent advances in membrane-integrated reactors, such as pervaporation and nanofiltration, which enable continuous water removal, shifting equilibrium and increasing conversion under milder conditions, are reviewed. Dual-functional membranes that combine catalytic activity with selective separation further enhance process efficiency and reduce energy consumption. Enzymatic systems using immobilized lipases present additional opportunities for mild and selective reactions. Future directions emphasize the integration of pervaporation membranes, hybrid catalyst systems combining biocatalysts and metals, and real-time optimization through artificial intelligence. Modular plug-and-play reactor designs are identified as a promising approach to flexible, scalable, and sustainable esterification. Overall, the interaction of catalyst development, membrane technology, and digital process control offers a transformative platform for next-generation ester synthesis aligned with green chemistry and industrial scalability.

## 1. Introduction

**Scheme 1:**
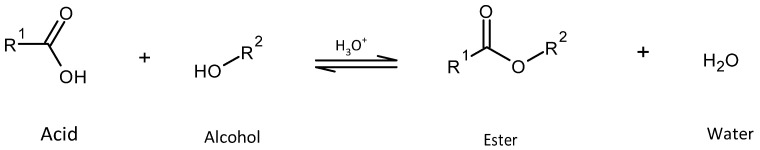
General reaction equation for an alcohol reacting with an acid to an ester and water.

Esterification is one of the most significant chemical reactions in the industrial sector, playing a crucial role in the production of plastics, pharmaceuticals, biofuels, surfactants, and flavorings. Chemically, esterification involves the reaction between a carboxylic acid and an alcohol, resulting in the formation of an ester and water [1]. This reaction is typically reversible and requires catalysts to achieve sufficient reaction rates. Catalysts accelerate the reaction by facilitating proton transfer, thereby enhancing the electrophilicity of the carbonyl carbon and enabling nucleophilic attacks by an alcohol. Scheme 1 illustrates the general reaction equation for esterification. 

While several reviews focus individually on catalysts, membranes, or reactor technologies, an integrative framework that evaluates their interplay under continuous conditions, especially with regard to stability, scalability, and process integration remains largely absent. This review aims to bridge this gap [2].

Despite extensive research, energy-intensive batch processes utilizing homogeneous acid catalysts remain predominant. These processes involve substantial operating costs, high-energy consumption, and environmental issues caused by corrosive byproducts. Concurrently, stricter environmental regulations such as the European Union (EU) Green Deal, which mandates a 55% reduction in CO_2_ emissions by 2030 and the Registration, Evaluation, Authorisation and Restriction of Chemicals (REACH) regulation, limiting hazardous chemical usage, are exerting additional pressure on the chemical industry [3]. Addressing these challenges requires innovative process intensification strategies that align environmental sustainability with economic competitiveness [4].

Classical esterification methods typically involve elevated temperatures, prolonged reaction times, and inherent thermodynamic limitations. Traditional water removal by distillation often needs temperatures of up to 250 °C, resulting in significant energy consumption [5]. Moreover, the use of strong mineral acids like sulfuric acid can severe corrosion issues and generate undesirable side-products. Thus, there is a growing necessity to develop sustainable alternatives that offer greater energy efficiency and more selective reaction pathways [6].

Membrane separation technologies, such as pervaporation, present a particularly promising solution by selectively removing reaction water at significantly lower temperatures (50–80 °C), potentially reducing energy requirements by up to 50%. Additional innovative membrane-based processes, including nanofiltration, electrodialysis, and membrane distillation, provide versatile solutions adaptable to diverse industrial applications [6].

This review systematically evaluates recent developments in membrane separation techniques, continuous reactor systems, and sustainable catalytic processes. It specifically investigates pervaporation, nanofiltration, and electrodialysis technologies (Section 4 and Section 5) for selective water removal and process optimization. Additionally, it provides a comprehensive comparison of homogeneous, heterogeneous, and enzymatic catalysts regarding stability, selectivity, and recyclability (Section 2). The economic feasibility and scalability of these technologies for industrial applications are also analyzed (Section 7) [7,8].

Despite the increasing number of publications on process intensification, a systematic integration of catalytic, separation, and reactor design aspects into a unified framework remains largely absent. Although many studies explore either catalytic systems, membrane technologies, or reactor designs individually, few provide a comparative evaluation of their combined application under continuous operation. This includes the compatibility of dual-functional materials, the durability of integrated membrane–catalyst systems, and their impact on industrially relevant process intensification (Section 6 and Section 7).

In contrast to previous reviews, this review emphasizes continuous processes and modern membrane separation technologies. It offers an integrative analysis of technological, economic, and ecological factors essential for industrial scale-up. The primary objective is to highlight the practical potentials and challenges associated with new process intensification methods, emphasizing their significance for sustainable and efficient chemical production [5,9].

## 2. Acid Catalysts for Esterification Reactions

This chapter explores various catalytic approaches to esterification, with a focus on three main categories: homogeneous catalysts, heterogeneous catalysts, and enzymatic catalysts. These catalysts play a crucial role in controlling reaction kinetics, product selectivity, and overall process efficiency. That is particularly relevant for industrial applications.

This section is confined to acidic and enzymatic catalyst systems, intentionally excluding basic catalysts due to their lesser relevance within the defined application context. This is based on the greater industrial and scientific relevance of acidic and enzymatic systems for esterification reactions, particularly in the synthesis of fine chemicals and bio-based products. The aim of this chapter is to provide a comprehensive overview of recent advancements and challenges in catalytic esterification using these systems. Both well-established methodologies and emerging strategies with the potential to further enhance the efficiency and sustainability of esterification reactions will be discussed.

### 2.1. Homogeneous Acid Catalysts

**Scheme 2:**
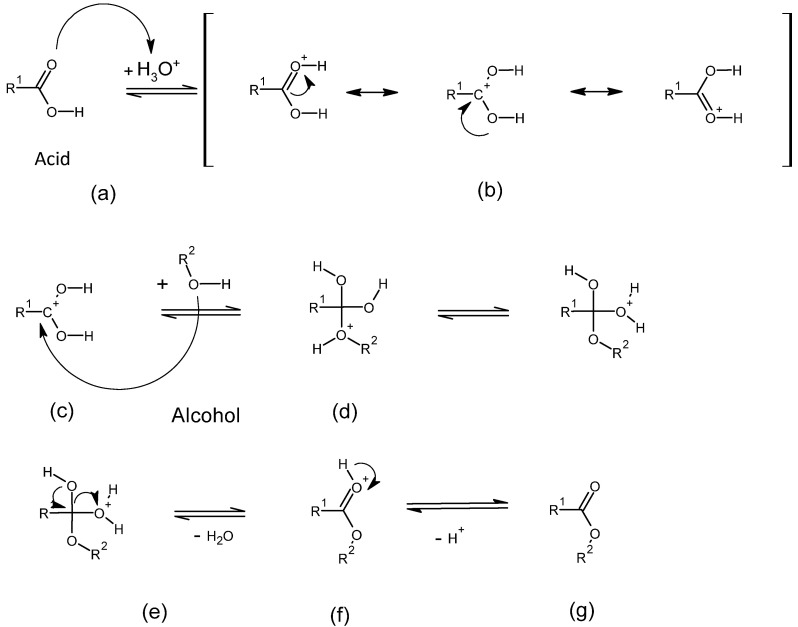
Acid-catalyzed esterification mechanism. Protonation of the carboxylic acid (**a**,**b**) activates the carbonyl for nucleophilic attack by the alcohol (**c**), forming a tetrahedral intermediate (**d**). After proton transfer and water elimination (**e**), the ester is formed via deprotonation (**f**,**g**).

Homogeneous acid catalysts play a central role in industrial esterification and transesterification processes, significantly accelerating the reaction through targeted protonation. Scheme 2 illustrates the reaction mechanism of an esterification reaction. Chemically, the reaction initially involves protonation of the oxygen atom in the carbonyl group of the carboxylic acid by a Brønsted acid (a) [10]. This protonation makes the carbonyl carbon more electrophilic (b), facilitating a nucleophilic attack by the alcohol’s oxygen atom (c). This attack leads to the formation of a tetrahedral intermediate (d), which subsequently undergoes an intramolecular proton transfer. Water is eliminated (e), and this results in ester formation (f, g) [11].

The efficiency of these catalysts depends on several factors, including their acidity, chemical and thermal stability under specific reaction conditions, selectivity towards desired products, and their potential for recovery and reuse [12,13].

Despite their widespread industrial use, homogeneous acid catalysts are associated with several significant drawbacks. Their non-recoverability leads to substantial waste generation and necessitates extensive downstream purification steps. Furthermore, their corrosive nature imposes strict material requirements on processing equipment, increasing capital and maintenance costs. Environmental regulations increasingly restrict the use of strong mineral acids, particularly when used at scale, due to disposal challenges and safety concerns [14].

In practice, various types of homogeneous acid catalysts are employed in industrial processes, including strong mineral acids such as sulfuric acid (H_2_SO_4_), hydrochloric acid (HCl), or perchloric acid (HClO_4_), as well as organic acids and ionic liquids (ILs). Ionic liquids have gained increasing importance due to their customizable acidity, high thermal stability, and recyclability [15]. Table 1 lists selected homogeneous acid catalysts along with their characteristic properties, typical applications, and achievable yields.

In Table 1, ionic liquids demonstrate remarkable thermal stability and flexibility in acidity tuning, with yields comparable to mineral acids, while offering potential for recyclability. However, most studies lack information on experimental replicability, making direct comparison challenging. The “Replicability” column provides qualitative insight into the consistency and transparency of the reported data, addressing this gap.

The esterification reaction is reversible and typically follows second-order kinetics, where concentrations of both the carboxylic acid and alcohol significantly affect reaction rates. Higher temperatures enhance the reaction rate but can also lead to evaporative losses of reactants and side reactions. Particularly strong acids like sulfuric acid, due to their higher protonation ability, show lower activation energies, resulting in faster reaction kinetics [24,25].

In real industrial processes, non-ideal conditions are frequently encountered, for example, high reactant concentrations, the use of polar solvents, or pronounced intermolecular interactions. Under such conditions, a substance’s reactivity no longer directly corresponds to its concentration, as assumed in ideal systems. To describe this deviation, so-called activity coefficients are introduced. These coefficients account for how strongly the behavior of a component differs from its idealized form. To calculate activity coefficients, various thermodynamic models are applied. Among the most widely used are:

- UNIFAC (Universal Functional-group Activity Coefficient model), which estimates activity based on group contributions within the molecule.
- UNIQUAC (Universal Quasi-Chemical model), which considers molecular size, shape, and local interactions.
- NRTL (Non-Random Two-Liquid model), which assumes non-random molecular distribution and is particularly useful for highly polar systems.

These models enable more accurate prediction of chemical equilibria and reaction kinetics, especially under conditions of high concentration or in the presence of polar or associating solvents. As such, they represent essential tools for the realistic modeling and optimization of esterification reactions in industrial applications [26].

The disadvantages associated with classical homogeneous acids, notably challenging separation, corrosion problems, and environmental impact. Those points have driven extensive research into sustainable alternatives. Ionic liquids (IL), especially Brønsted-acidic ILs such as 1-(4-sulfonic acid)butylpyridinium hydrogen sulfate, offer low volatility and good recyclability. A significant challenge with these catalysts is their sensitivity to water, which reduces catalytic activity due to solvation of acidic groups. Immobilization on mesoporous materials such as silica or magnetic nanoparticles can significantly mitigate these issues by enhancing the stability of acidic sites and simplifying catalyst recovery [10,27].

To enhance catalytic performance, reactive additives are often used. Such catalysts exploit the complementary effects of Brønsted and Lewis acidity to improve conversion and selectivity. Similarly, dehydrating agents like molecular sieves remove water formed during the reaction, shifting equilibrium towards product formation. Polar aprotic solvents, especially dimethyl sulfoxide (DMSO), increase solubility and reactivity of starting materials by facilitating nucleophilic attacks [28,29].

Hybrid catalysts that combine Lewis-acidic and Brønsted-basic properties offer additional optimization potential. Titanium-based aminotriphenolate complexes are particularly notable in this context. These complexes enable efficient proton transfer and intramolecular preorganization of substrates through hydrogen bonding, thereby enhancing catalytic activity and selectivity. Such hybrid catalysts combine advantages of homogeneous and heterogeneous systems, presenting promising developments for sustainable and efficient catalytic processes [13,30].

### 2.2. Heterogeneous Acid Catalysts

The use of heterogeneous catalysts offers several advantages over homogeneous systems, including simplified product separation, catalyst reusability, and reduced waste generation, thus contributing to more sustainable chemical processes. Among these, heterogeneous acid catalysts (SACs) have emerged as particularly promising materials for esterification. These systems are based on solid supports that are physically or chemically modified with acidic functional groups to enhance their catalytic activity [31,32].

The fundamental mechanism of heterogeneous esterification using porous catalysts involves a sequence of mass transport and surface reaction processes. Initially, the reactants must pass through the external boundary layer surrounding the catalyst particles, a step commonly referred to as film diffusion. Once this barrier is overcome, the reactants enter the porous structure of the catalyst via pore diffusion. Inside these pores, the molecules adsorb onto the internal surface of the material, where the actual esterification reaction takes place at the catalytically active sites. After the reaction, the product molecules desorb from the surface and migrate out of the pore network, eventually passing through the boundary layer and reentering the bulk phase of the reaction mixture [13,33,34].

The reactivity of such systems is governed largely by chemisorption at specific active sites, such as edges, defects, and corners, where reactants form covalent interactions with the surface. Depending on the substrate and surface chemistry of the catalyst, adsorption may occur either associatively (intact molecules) or dissociatively (fragmentation of the molecules). A clear distinction from physisorption, which involves only weak van der Waals interactions, is essential for understanding the nature of catalytic activity in these systems. In heterogeneous catalysis, once chemisorption has occurred, the reaction mechanism can typically be described by one of two classical models: the Langmuir–Hinshelwood or the Eley–Rideal mechanism [35,36,37].

While both Langmuir–Hinshelwood and Eley–Rideal models offer conceptual frameworks for describing esterification kinetics on solid catalysts, their direct experimental validation remains challenging. Most studies rely on fitting reaction data to derive apparent kinetic parameters, without independently confirming adsorption equilibria or surface coverages. Thus, these models should be interpreted as theoretical constructs that aid in qualitative understanding, rather than universally confirmed mechanistic truths.

In the Langmuir–Hinshelwood mechanism, both reactants are first adsorbed onto the catalyst surface and then undergo a surface-mediated reaction. This pathway assumes competitive adsorption and is particularly relevant when the catalyst surface features high coverage and when adsorption equilibria significantly influence the reaction rate. It is common in systems where both reactants have comparable affinities for the surface [38,39].

In contrast, the Eley–Rideal mechanism involves only one reactant being adsorbed on the catalyst surface, while the other reacts directly from the gas or liquid phase. This mechanism is more likely when one reactant either has a low adsorption affinity or is present at low concentration. It can become dominant in systems where steric hindrance or strong site specificity restrict the co-adsorption of multiple reactants [40].

**Figure 1:**
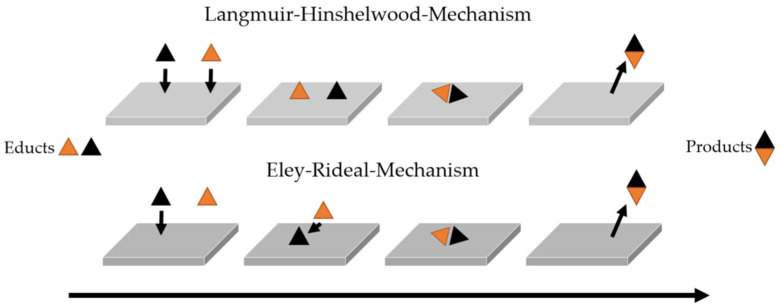
Schematic representation of the Langmuir–Hinshelwood and Eley–Rideal Mechanisms in heterogeneous catalysis. In both cases, a black triangle (educt) and an orange triangle (educt) react to form a black–orange product [37].

These mechanistic models are grounded in theoretical considerations and idealized surface behavior. The actual dominant pathway depends strongly on surface coverage, reactant concentrations, adsorption energies, and temperature. Understanding which mechanism applies is crucial for rational catalyst design and the development of reliable kinetic models in chemisorption-driven systems [26,38]. Figure 1 schematically illustrates both pathways, emphasizing the distinct sequence of adsorption and reaction steps [37].

In the following section, various heterogeneous catalyst systems will be reviewed, focusing on their structural characteristics, mechanistic behavior, and application potential in esterification reactions. Particular emphasis is placed on catalyst material class, acidity, porosity, and environmental compatibility.

#### 2.2.1. Ion Exchange Resins

Ion exchange resins are synthetic polymeric materials functionalized with acidic groups that serve as catalytic centers. The most important type of ion exchange resins used in esterification reactions are strongly acidic cation exchange resins, which typically carry sulfonic acid groups (-SO_3_H) covalently bound to a polymer matrix, often based on polystyrene cross-linked with divinylbenzene (DVB) [41,42].

These -SO_3_H groups exhibit strong Brønsted acidity, enabling the resin to efficiently donate protons to initiate acid-catalyzed reactions such as esterification. The acidic sites are immobilized on an insoluble matrix, which allows heterogeneous catalysis with the advantages of easy separation and recyclability [43].

Amberlyst 15, a widely used sulfonated polystyrene-divinylbenzene resin, is particularly effective for the esterification of free fatty acids (FFAs) due to its high catalytic activity under moderate thermal conditions (typically up to ~140 °C) [44]. For higher temperatures, Amberlyst 70 is preferred due to improved stability and tolerance up to 190 °C. [45]. Table 2 lists selected ion exchange resins catalysts along with their thermal stability, functional groups, typical applications, and achievable yields. 

Amberlyst 15 and 70, both based on sulfonated polystyrene-divinylbenzene, offer high acidity and thermal stability, with Amberlyst 70 being more suitable for elevated temperature processes. Nafion™ NR50, a perfluorinated resin, combines strong Brønsted acidity with exceptional chemical resistance, ideal for harsh conditions. Experimental replicability isoften not reported in detail (cf. Section 2.1). Some studies indicate methodological robustness, though often without quantitative validation.

Nafion™ NR50, represents a perfluorinated sulfonic acid resin, structurally based on a polytetrafluoroethylene (PTFE) backbone with perfluoroalkyl side chains terminated by -SO_3_H groups. It combines exceptional chemical and thermal stability with high acidity and is often employed in demanding industrial syntheses where resistance to aggressive solvents or oxidative environments is essential. While its catalytic performance is high, the cost and processing complexity of Nafion™ limit its use to specialized applications [5,46].

Despite their many advantages, ion exchange resins also face certain limitations that constrain their broader industrial application. Their performance is often diffusion-limited, particularly in reactions involving bulky molecules or highly viscous media, due to restricted accessibility of active sites within the polymer matrix. Furthermore, ion exchange resins can exhibit sensitivity to long-term thermal or mechanical stress, leading to swelling, shrinkage, or gradual deactivation under harsh operating conditions. Catalyst fouling and pore blockage by high-molecular-weight compounds or byproducts may reduce activity over time, necessitating periodic regeneration or replacement. Finally, while some resins offer recyclability, the number of effective reuse cycles is finite and strongly dependent on the chemical environment [47,48].

#### 2.2.2. Zeolithes

Zeolites are a structurally versatile class of solid acid catalysts with significant importance in esterification chemistry. Their thermal stability, tunable acidity, and defined porosity make them ideal candidates for acid-catalyzed transformations. Among them, beta zeolites (BEA-Type) have shown superior performance, especially in the esterification of carboxylic acids, outperforming structures such as Faujasit (FAU) (Y-Type) and Mobil-type five (MFI) (ZSM-5) due to their favorable pore systems and acidity profiles [49].

A key challenge in the use of microporous zeolites is the diffusional limitation for bulkier substrates. This has led to the development of hierarchical zeolites, which integrate mesoporosity into the microporous framework. Beta-Zeolites (BEA) modified via desilication and subsequent acid washing exhibit increased mesopore volume and improved accessibility to Brønsted acid sites, significantly enhancing yields and turnover frequencies in reactions with large molecules like benzofuran [50].

In the context of bio-oil upgrading, esterification plays a pivotal role in reducing the corrosivity of organic acids. Modified H-beta zeolites, treated with organic acids such as malic, oxalic, or tartaric acid, allow for simultaneous dealumination and realumination, (i.e., controlled reintroduction of Al^3+^ to regenerate Brønsted acid sites) thereby fine-tuning acid site distribution [51,52]. Furthermore, natural zeolites such as mordenite and clinoptilolite can be significantly improved by multi-stage dealumination, leading to very high Si/Al ratios and large surface areas, making them suitable for esterification in complex media [53].

Lastly, hierarchical zeolite Y (FAU) benefits from sequential acid and base treatments. Optimized acid washing enhances Brønsted acidity while maintaining structural integrity, further boosting its catalytic performance [49,50]. Table 3 provides an overview of selected zeolite catalysts and their performance characteristics in esterification reactions.

These findings underline the value of tailored structural and acidic modification of zeolites for maximizing performance in esterification, particularly for bulky substrates, complex feedstocks, or continuous industrial processes. As previously noted for other catalyst classes, the reproducibility of these findings is difficult to assess.

Despite their many advantages, zeolites also present limitations that must be carefully considered in catalyst design. The intrinsic microporosity of conventional zeolites restricts the accessibility of active sites for large or multifunctional molecules, often leading to diffusion-controlled reaction rates. While hierarchical structuring can mitigate these issues by introducing mesopores, the synthesis of such materials can be complex and insufficiently reproducible at scale. Additionally, strong acidity combined with limited pore volume may lead to rapid deactivation due to coke formation or pore blockage in reactions involving heavy organic species. These factors necessitate a balanced optimization between acidity, pore size distribution, and structural integrity for practical applications [53,55].

#### 2.2.3. Mesoporous Materials

While zeolites continue to be foundational in solid acid catalysis, the development of tailored mesoporous materials has opened new avenues for esterification reactions especially under demanding conditions such as high molecular weight substrates, water-rich systems, or supercritical media. Compared to conventional microporous zeolites, these materials exhibit significantly reduced diffusional limitations due to their larger and more accessible pore structures. These materials combine high surface areas, controlled pore architectures, and customizable acid functionalities, making them highly attractive for fine-tuning catalytic performance in both batch and continuous processes.

Among these, sulfonated mesoporous polymers such as Sulfonated, ordered mesoporous polymer doped with p-toluenesulfonic acid (OMP-TsOH), synthesized using Santa Barbara Amorphous-15 (SBA-15) or Mobil Composition of Matter-48 (MCM-48) as hard templates, represent a promising class of materials. Due to their exceptionally high acid site densities (up to 4.9 mmol/g) and large surface areas (up to 1400 m^2^/g), they enable efficient catalysis even for bulky substrates like oleic acid [56,57,58].

The incorporation of heteropoly acids (HPAs), such as tungstophosphoric acid (TPA) or phosphotungstic acid (HPW), into mesoporous silica matrices further enhances catalytic activity while preserving heterogeneity. For instance, TPA@MCM-48 has been applied in the esterification of glycerol with acetic acid, yielding up to 50% diacylglycerol (DAG) and 30% triacylglycerol (TAG), with good reusability over several cycles. Similarly, HPW-supported systems demonstrate high activity even under supercritical CO_2_ conditions, where the longer chain length of fatty acids correlates with increased conversion efficiency [59].

In another approach, ionic liquids (ILs) have been immobilized onto metal-doped mesoporous supports such as Fe-SBA-15, enabling dual acid functionality (Brønsted and Lewis) in a recyclable solid form. The resulting IL/Fe-SBA-15 catalyst achieved 81.4% conversion of oleic acid in methanol under mild condition [60,61].

Organically bridged periodic mesoporous organosilicas (PMOs) represent a further innovation in water-tolerant esterification catalysts. These materials retain their structural integrity and acidity even in the presence of polar byproducts such as water and glycerol, making them ideal for biodiesel applications [56].

Another promising material is sulfated TiO_2_ dispersed in SBA-15 (S-TiO_2_/SBA-15). This hybrid solid acid catalyst achieved nearly 95% conversion of waste cooking oil, demonstrating high reactivity, stability, and reusability. Its robustness and scalability position it is a strong candidate for industrial esterification processes [62].

Recent work has also highlighted the catalytic potential of Functionalized Silica Material-16 (FSM-16)-SO_3_H, a sulfonated folded-sheet mesoporous silica prepared via sol-gel synthesis and post-functionalization with chlorosulfonic acid. The material features a BET surface area of 634 m^2^/g, a pore diameter of ~6 nm, and a combination of Brønsted and Lewis acid sites, as confirmed by pyridine- (Fourier transform infrared spectroscopy) FTIR spectroscopy. Applied in multicomponent condensations such as the Hantzsch reaction, FSM-16-SO_3_H achieved up to 93% product yield and retained its activity across multiple cycles—highlighting its broader potential in acid-catalyzed organic transformations, including esterifications [63].

Lastly, Chimie des Matériaux Inorganiques-10 (CMI-10), a mesoporous aluminosilicate with a Si/Al = 1, deserves special attention due to its remarkable hydrothermal stability. Its acid site architecture, hydrophilic surface, and long-range mesoscopic order suggest high potential for future application in aqueous-phase esterification and biomass upgrading chemistry. Table 4 provides an overview of selected mesoporous polymer- and silica-based catalysts and their performance characteristics in esterification reactions [64].

As previously discussed for other catalyst classes, reproducibility is also inconsistently addressed for the materials listed in Table 4. While SBA-15 and MCM-48-20HPW were tested under varying conditions, repeated trials under identical settings are missing. IL/Fe-SBA-15 is the only example with data evaluated at a 95% confidence level. For SBA-15-PrSO_3_H, FSM-16-SO_3_H, and CMI-10, reusability or catalyst variations were explored, but systematic replicability was not evaluated. This recurring issue limits cross-study comparability.

Despite their excellent catalytic properties, the synthesis of mesoporous polymer and silica-based materials often involves multiple templating, functionalization, and calcination steps, which can increase production cost and limit large-scale applicability.

#### 2.2.4. Metal Oxides

While metal oxides are well-established catalysts for fatty acid esterification, their application is expanding to low-molecular-weight carboxylic acids (e.g., acetic, lactic, and hydroxypropionic acid) and to upgrading bio-oil mixtures with high acid and water content. These systems require not only catalytic activity but also water tolerance, suitable acid site distribution, and reusability. Oxides like TiO_2_, ZrO_2_, or Al_2_O_3_, especially in sulfated or modified forms, offer the flexibility to meet these demands. Tailored control over Brønsted vs. Lewis acidity and surface architecture (e.g., via sol-gel or Metal-Organic Framework (MOF) templating) enables effective catalysis under aqueous or mild conditions [65,66].

A study by Leahy et al. used sulfated ZrO_2_-TiO_2_ for the esterification of acetic acid with ethanol, simulating bio-oil upgrading. A composition with 50 wt% ZrO_2_ achieved 93.7% conversion at 100 °C, attributed to strong Brønsted acidity from sulfate groups (confirmed by FTIR and NH_3_-TPD) [66,67,68].

Zhang et al. developed a permanent white (PW-TiO_2_) catalyst by immobilizing H_3_PW_12_O_40_ on a porous TiO_2_ matrix (derived from MOF-125(Ti)), yielding 90.5% conversion of oleic acid. Despite oleic acid as the substrate, its MOF-derived porosity and accessible Brønsted sites make it suitable for smaller, polar acids under mild, aqueous conditions. The catalyst retained 74% activity after six cycles [67,69].

Ropero-Vega et al. synthesized sulfated titanium via sol-gel and ammonium sulfate impregnation. The material exhibited Brønsted and Lewis acidity, achieving 82.2% conversion of oleic acid at 80 °C with full selectivity. Its acid density and stability suggest potential for short-chain acid esterification [70,71].

Table 5 summarizes the most relevant systems and their performance in esterification of low-molecular-weight carboxylic acids and complex bio-oil feedstocks.

Table 5 presents selected metal oxide-based catalysts applied to the esterification of low-molecular-weight acids and bio-oil components. Sulfated mixed oxides (e.g., SO_4_^2−^/ZrO_2_-TiO_2_ and ZrO_2_/SO_4_) demonstrate strong Brønsted acidity and achieve high conversions, though often under optimized conditions. PW-TiO_2_ (MOF-derived) combines high activity with good reusability, while CaO/Al_2_O_3_ achieves excellent conversion with dual acid-base functionality. As in previous cases, reproducibility data (e.g., standard deviations or repeated trials) are mostly lacking, with the exception of PW-TiO_2_, where recyclability was explicitly tested over multiple cycles.

These findings underscore the potential of metal oxide catalysts. Whether sulfated, heteropolyacid-modified, or MOF-derived, broader esterification chemistry beyond long-chain fatty acids. Nonetheless, challenges such as synthesis complexity, limited recyclability in harsh media, and sensitivity to water-induced deactivation must still be addressed to enable robust industrial deployment. Their tunable acid-base properties and structural robustness make them ideal for transforming fermentation-derived platform chemicals, bio-oil acids, or aqueous reaction mixtures into valuable esters. Future developments in catalyst design and substrate scope will further unlock their potential in green and sustainable chemical processes.

#### 2.2.5. Metall–Organic Framework

Metal–organic frameworks (MOFs) have emerged as a versatile class of porous crystalline materials whose modular composition enables rational design of catalytically active architectures. Their hybrid nature enables precise functionalization either at the metal nodes (coordinatively unsaturated sites, Lewis acids), on the organic linkers (Brønsted acid groups), or within the pore cavities through post-synthetic modification or guest encapsulation, such as with polyoxometalates (POMs). These features make MOFs excellent platforms for catalytic esterification and selective oxidation [72].

A representative example is S-MOF-101, synthesized via a one-pot introduction of sulfonated dicarboxylate linkers. The SO_3_H groups, covalently bound to the terephthalic acid linker, act as strong Brønsted acid sites. These activate carboxylic acids by hydrogen bonding to the carbonyl oxygen, increasing its electrophilicity towards nucleophilic attack by alcohols. The proximity of active sites and high pore accessibility promotes effective substrate diffusion and interaction, even for bulkier acids. In esterification reactions, S-MOF-101 demonstrates high turnover and reusability over five cycles without measurable activity loss [73].

MOF-101(Cr)-SO_3_H was synthesized in a one-pot approach using chromium salts (either CrO_3_ or Cr(NO_3_)_3_·9H_2_O) and 2-sulfoterephthalic acid monosodium salt as the functionalized linker. Among these, the use of HCl led to a material with superior textural and acidic properties, featuring higher crystallinity, larger surface area, and more pronounced Brønsted acidity. The sulfonic acid moieties introduced via the linker remained catalytically active after thermal treatment. In the esterification of cyclohexene with formic acid, used here as a model system relevant to in situ cyclohexanol synthesis, the HCl-mineralized material reached a conversion of nearly 64% with excellent selectivity (>97%) for the ester product. FTIR spectroscopy with pyridine adsorption revealed that Brønsted acid sites were predominant compared to Lewis-acidic Cr(III) centers. Furthermore, catalytic activity and structural integrity remained stable across three consecutive reaction cycles [74,75].

Polyoxometalates (POMs) immobilized within MOFs represent a strategy to introduce multi-electron redox-active Brønsted acid sites. In MOF-101(PW_12_), the Keggin-type anion [PW_12_O_40_]^3−^ is confined within the supercages of MOF-101 via diffusion and electrostatic stabilization. The resulting hybrid benefits from the POM’s inherent acidity and redox functionality, while the MOF matrix prevents leaching and provides shape-selective access to substrates [71,73]. The versatility of POM-MOF composites is further demonstrated in the PWx/MOF-101 systems, where different POM loadings (x = 4, 12) were examined. Higher loadings enhanced the number of accessible acidic centers, but excessive amounts risk pore blockage or reduced dispersion. Optimal systems (ca. 10–12 wt%) balanced high Turnover Frequency (TOF) with structural integrity [75,76].

The following Table 6 summarizes selected MOF-based catalysts used in esterification and oxidation reactions:

Table 6 presents two sulfonated MOF-based catalysts applied to esterification. S-MOF-101 exhibits excellent activity (99% conversion) toward monocarboxylic acids, enabled by uniformly distributed Brønsted acid sites. MOF-101(Cr)-SO_3_H, synthesized with HCl as mineralizer, achieves 63.9% conversion of oleic acid to methyl oleate, demonstrating applicability in biodiesel synthesis.

These findings highlight the chemical flexibility of MOFs in solid acid catalysis. Their defined pore architecture facilitates substrate access and product removal, while engineered acid functionalities provide tunable activation pathways for carboxylic acids and olefins alike. Nevertheless, the hydrothermal and mechanical stability of many MOFs especially under industrially relevant reaction conditions remains a significant concern [77].

In addition, the synthesis of functionalized MOFs often involves multi-step procedures, expensive ligands, and long reaction times, which may hinder scalability. However, the reusability and hydrolytic stability of MOF catalysts remain major advantages over homogeneous or amorphous solid acids, especially in water-rich or oxidative systems. As research progresses, dual-functional MOFs, framework-defect engineering, and enzyme-MOF hybrids will likely shape the next generation of catalytic materials for biomass valorization and green chemical synthesis [78].

### 2.3. Continuous Enzymatic Esterification

The development of efficient and sustainable processes for ester synthesis, particularly for applications in immobilizer production, lubricant formulation, and platform chemicals has increasingly focused on biocatalytic strategies. Among these, continuous enzymatic esterification has emerged as a promising alternative to traditional chemical catalysis. 

Lipases are serine hydrolases that catalyze the hydrolysis of ester bonds under aqueous conditions but are equally capable of catalyzing ester synthesis and transesterification in low-water or non-aqueous environments. In esterification reactions, lipases function as biocatalysts by facilitating the nucleophilic attack of an alcohol on the carbonyl carbon of a carboxylic acid, leading to ester formation with water as a byproduct. Their active site typically contains a catalytic triad (serine, histidine, and aspartate or glutamate) embedded within a hydrophobic pocket, which provides high substrate specificity and regioselectivity [79].

Mechanistically, the esterification proceeds via an acyl-enzyme intermediate: the carboxylic acid first forms a covalent bond with the serine hydroxyl, creating an acylated enzyme. This intermediate is subsequently attacked by the alcohol, leading to ester release and regeneration of the free enzyme. The reaction can be operated under mild conditions (30–60 °C, atmospheric pressure), making it highly suitable for thermolabile or sensitive substrates. Additionally, lipases tolerate a wide range of organic solvents, particularly hydrophobic ones, which enhance substrate solubility and drive the equilibrium toward ester formation by reducing water activity [80].

Central to the success of the esterification reaction are two synergistic concepts: (1) the immobilization of lipases [81] on tailored carrier materials to enhance enzyme stability and recyclability, and (2) the integration of pervaporation, [82] a membrane-based separation technology that removes inhibitory byproducts, thereby intensifying the process. Together, these strategies offer a pathway toward highly selective, mild, and economically viable continuous processes.

#### Selection and Evaluation of Carrier Materials for Immobilized Enzymes

Immobilizing enzymes on solid supports extends their lifetime and facilitates their recovery, but the benefits go far beyond reusability. The physicochemical interactions between the enzyme and its carrier significantly influence the conformation, catalytic activity, and stability of the biocatalyst under process conditions. Consequently, the selection of the carrier material is not merely a mechanical design choice but a critical aspect of catalyst engineering [83].

Carrier materials can be broadly classified according to their immobilization mechanism—such as physical adsorption, ionic binding, or covalent attachment—each of which influences enzyme behavior in unique ways. Hydrophobic polymeric materials, like modified acrylic resins (e.g., Novozym^®^ 435), are widely used for their strong affinity to the hydrophobic patches of lipases. These supports favor enzyme adsorption without significant structural deformation, making them well suited for non-aqueous systems and organic solvents often encountered in esterification reactions. Due to their chemical inertness and mechanical resilience, they are ideal for both batch and packed-bed flow reactors. Nonetheless, purely hydrophobic carriers may suffer from limited applicability in aqueous or biphasic systems, where desorption or local aggregation can occur [84].

Covalent immobilization, by contrast, offers superior resistance to leaching and mechanical degradation. Functional groups such as epoxides, aldehydes, or carboxyls can be introduced on silica, epoxy-silicate networks, or natural polymers like chitosan to provide stable binding sites for enzyme anchoring. This method is especially advantageous in microreactor environments, where high shear rates and long operational times demand robust catalyst materials. However, the covalent approach often requires complex and multi-step surface modifications, and irreversible binding may hinder enzyme regeneration or recycling [85,86].

Magnetic carriers, such as superparamagnetic nanoparticles, add another dimension of process flexibility by allowing external manipulation and easy catalyst recovery. For example, Guo et al. demonstrated that Candida rugosa lipase immobilized on magnetic hydrophobic microspheres achieved more than double the activity of its free counterpart and could be rapidly recovered via magnetic separation-making it particularly appealing for modular or multi-stage continuous systems [85,86].

Porous materials such as mesoporous silica, polyurethane foams, or ceramic scaffolds are often engineered to combine high surface area with tailored functionalization. Their internal pore structures can provide protective microenvironments for the enzyme while maintaining high substrate accessibility. When integrated into hybrid modules, these carriers not only immobilize the enzyme but also function as structural supports for membranes, enabling one-step catalysis-separation systems [76].

Table 7 summarizes a selection of lipases, carriers, and reactor formats commonly encountered in the literature.

Table 7 highlights various immobilized lipases used in esterification, covering different carriers and reactor formats. Magnetic microspheres (e.g., for Candida rugosa) enable fast separation and enhanced activity, while polymer- and membrane-based systems (e.g., Lipozyme Thermomyces lanuginosus and Rhizomucor miehei (TL/RM IM)) offer high efficiency in continuous or microreactor setups. Novozym 435 remains a versatile standard in organic media. Hybrid supports like chitosan or mesoporous silica show high stability and compatibility with integrated systems. As in earlier cases, detailed replicability data are limited.

The integration of immobilized lipases and pervaporation offers a compelling route for process intensification in enzymatic esterification. However, challenges remain: enzyme activity must be retained over extended cycles, membrane fouling must be mitigated, and operational stability must be ensured under dynamic flow conditions. Additionally, scale-250 up of combined enzymatic-membrane systems remains difficult due to complex fluid dynamics and fouling behavior, which are not always predictable from lab-scale models. Despite these challenges, recent advances have significantly closed the performance gap between enzymatic and chemical catalysis in continuous systems [89].

Despite these challenges, recent advances in enzyme immobilization and hybrid reactor design have narrowed the performance gap between enzymatic and chemical catalysis. For example, reusability studies report that immobilized lipases can retain over 70% of their activity after eight cycles, making continuous enzymatic processing increasingly viable at an industrial scale [90].

Looking forward, we expect further miniaturization of integrated systems (e.g., lab-on-chip devices), modular system architectures with plug-and-play reactor-separator units, and the incorporation of real-time process analytics to enable automated control and optimization. Such innovations could establish continuous enzymatic esterification not only as a green alternative to acid catalysis but as a robust, scalable solution for future biomanufacturing. Nonetheless, the high cost of enzymes, dependency on careful environmental control (e.g., pH, temperature), and regulatory constraints for biocatalytic processes remain barriers to full industrial adoption [91]. To our knowledge, a systematic comparison of catalytic systems, across homogeneous, heterogeneous, and enzymatic domains, within integrated membrane or intensified reactor systems remains underrepresented in literature. The following chapters aim to provide such a comparative perspective, linking material properties with reactor functionality and separation efficiency.

## 3. Structure-Property Relationships of Esters: Monoesters, Diesters, and Chain Length Effects

### 3.1. Monoesters vs. Diesters: Molecular Structure and Function

Monoesters are formed by the esterification of an alcohol with a carboxylic acid, resulting in the incorporation of a single acyl group into the molecular structure. This reaction is typically catalyzed by acidic catalysts or lipases, with enzymatic systems enabling enhanced selectivity in product formation. Kinetic studies indicate a Langmuir–Hinshelwood-type mechanism (Section 2.2), in which both adsorption and the surface reaction play key roles. The activation energy is approximately 100.4 ± 1.8 kJ/mol for the forward reaction and 118.3 ± 1.8 kJ/mol for the reverse reaction [92,93].

The physicochemical distinction between monoesters and diesters are fundamental. Monoesters exhibit higher polarity and a water affinity, rendering them more hydroscopic, more water soluble, and less thermally stable, with lower melting and boiling points. They are primarily used as emulsifiers in food, cosmetic, and pharmaceutical applications. Diesters, by contrast, are more lipophilic, less hygroscopic, more viscous. Their ability to form network-like structures and worm-like micelles leads to rheological viscosity maxima at around 40–50°C, as demonstrated by rheological measurements and small-angle X-ray scattering (SAXS). Rheological behavior is highly dependent on temperature, concentration, and the molecular geometry of the constituents. Functionally, diesters are important as plasticizers, lubricants, biolubricants, and phase change materials (PCMs). Notably, linear diesters are biodegraded significantly faster than branched analogs, underscoring their suitability for sustainable applications such as biolubricants or plasticizers. A detailed discussion of this aspect is provided in Section 3.3 [94,95].

These structural and functional differences are reflected not only in material properties but also in the underlying reaction kinetics. To describe the kinetics of monoester and diester formation, mechanistic models and equations that account for adsorption, reaction rate, and thermodynamic parameters can be applied, as outlined below [92,96].

The reaction rate r of a Langmuir–Hinshelwood mechanism can be expressed as follows [94]:

(1) $$r=\frac{k\cdot K_{A}\cdot C_{A}\cdot K_{B}\cdot C_{B}}{{\left(1+K_{A}\cdot C_{A}\cdot K_{B}\cdot C_{B}\right)}^{2}}$$

where k is the intrinsic rate constant, K_A_ and K_B_ are the adsorption coefficients, and C_A_ and C_B_ are the concentrations of reactants A and B.

The temperature dependence of k follows the Arrhenius Equation (2):

(2) $$k=A\cdot e^{-\frac{E_{a}}{RT}}$$

where A is the pre-exponential factor, E_a_ the activation energy, R the gas constant, and T the absolute temperature. Experimental studies report activation energies in the range of 50–120 kJ/mol, depending on catalyst, substrates, and solvent [91,93].

Diester formation involves an additional acylation step, making the reaction a sequential process:

(3) $$A+B\;\;\overset{k_{1}}{⇄}\;C+E\;\;\text{Monoester}$$

(4) $$C+B\;\;\overset{k_{2}}{⇄}\;D+E\;\;\text{Diester}$$

The temporal changes in concentrations are described by the following differential equations:

(5) $$\;\frac{d\left[C\right]}{dt}=k_{1}\left[A\right]\left[B\right]-k_{2}\left[C\right]\left[B\right]$$

(6) $$\frac{d\left[D\right]}{dt}=k_{2}\left[C\right]\left[B\right]$$

The selectivity between mono- and diester is largely governed by the ratio k_1_/k_2_, reaction time, and reaction conditions. Experimentally, diester formation can be favored by an excess of acylating agent or extended reaction times; conversely, monoester selectivity can be enhanced by lowering the temperature or by tuning catalyst activity [96,97,98,99].

Typical reaction conditions for monoester formation are in the range of 65–110 °C, employing 13–19 mmol of solid catalyst (TiP) per 25 mmol alcohol with azeotropic water removal (e.g., using toluene or cyclohexane). Diester synthesis generally requires higher temperatures (110–155 °C), longer reaction times (8–10 h), and higher catalyst loadings [93]. This understanding is pivotal for the targeted synthesis of the tailored design of mono- and diesters for specific industrial, pharmaceutical, and sustainable applications [92].

### 3.2. Effect of Alcohol and Fatty Acid Chain Length

The chain length of the alcohols and fatty acids used has a significant influence on the physical, chemical, and mechanical properties of the resulting esters. Short-chain alcohols such as methanol or ethanol yield volatile, low-viscosity esters that are generally highly water-soluble. In contrast, long-chain alcohols (C_12_-C_18_) lead to highly viscous, hydrophobic esters with pronounced lubricating properties and increased lipophilicity. Similar effects are observed with fatty acids: short-chain fatty acids (C_4_-C_8_), such as butyric acid, produce water-soluble esters with a characteristic, often pungent odor, whereas long-chain fatty acids (C_12_-C_22_), such as stearic acid, yield lipophilic esters with film-forming and barrier-enhancing properties [96,100].

From a kinetic perspective, chain length has a pronounced impact on the reaction rate. Longer alkyl chains result in higher activation barriers and diffusion resistances, leading to extended reaction times and often necessitating evaluated temperatures or specified catalysts. These effects occur in both monoester and diester formation but are more pronounced in diesters due to their greater steric demand [92].

An interesting observation is that despite their more complex structure, diesters often exhibit lower crystallization activation energies compared to monoesters of similar chain length. These differences explain the faster crystallization rates and lower melting points observed for diesters. Thus, chain length not only affects the solubility and mechanical properties of esters but also influences their thermal and kinetic parameters—an important consideration in the targeted synthesis of esters for functional applications [93,101].

### 3.3. Structure-Dependent Material Properties and Applications

The structure of esters plays a decisive role in determining their material properties and, consequently, their potential applications. The hydrophilic–lipophilic balance (HLB) can be precisely tuned by adjusting the chain length and the number of acyl groups, a strategy commonly used in surfactant development. Typical examples include sorbitan esters or mono- and diglycerides. In sucrose esters, a high diester content leads to network-like supramolecular assemblies that transform into worm-like micelles upon heating, resulting in a viscosity maximum, an effect relevant for both food technology and pharmaceutical formulations [93,102].

Diesters derived from long-chain alcohols and fatty acids serve as bio-based plasticizers and lubricants that can replace environmentally critical phthalates. Diesters are also investigated as organic PCMs for thermal energy storage. In a comparative study, twelve diesters based on four dicarboxylic acids and three alcohols were synthesized with high purity and thermally characterized. While their melting points vary widely (−20 °C to 46 °C), most exhibited lower melting onset temperatures and higher supercooling tendencies than comparable fatty esters. Their enthalpies of fusion range from 92 J/g to 172 J/g, which is lower than typical linear saturated fatty esters (up to 200 J/g) [102,103,104].

In pharmaceutical applications, monoesters are preferred as carrier systems for lipophilic active ingredients due to their favorable solubility profile. In contrast, diesters function as depot formulations that enable prolonged drug release. Studies on astaxanthin esters showed a significantly higher oral bioavailability for monoesters, likely due to more efficient enzymatic cleavage and improved membrane permeability [93].

The biodegradation behavior of diesters is governed by key structural parameters, particularly chain length, linearity, and the degree of branching. Linear diesters with medium-length alkyl chains (C_8_–C_12_) undergo relatively rapid enzymatic hydrolysis. In contrast, increased molecular branching, especially near the ester moiety, leads to steric hindrance that impedes enzyme binding. Furthermore, long-chain diesters with highly hydrophobic backbones exhibit limited aqueous solubility and lower bioavailability, which significantly slows down microbial attack [105].

Experimental studies using respirometric assays and CO_2_ evolution tests confirm that linear saturated diesters (e.g., dioctyl adipate) show significantly higher biodegradation rates than their branched counterparts (e.g., bis(2-ethylhexyl) phthalate). The presence of terminal or internal branching can reduce degradation rates by more than 50%, depending on the branching topology and substitution pattern. These findings highlight the critical balance between functionality and environmental compatibility in ester design, especially for applications in biolubricants, plasticizers, and phase change materials, where both performance and end-of-life degradation must be optimized [105].

## 4. Process Intensification in Esterification—Technologies and Principles

Building on the understanding of structure–property relationships in esters, it becomes evident that optimizing esterification processes requires not only precise molecular design but also technological innovation at the process level. Process intensification systematically reengineers traditional reactor concepts to increase energy efficiency, minimize plant footprint, and reduce both capital and operational costs. Within esterification, continuous-flow reactor systems have gained growing importance [106].

A central principle of process intensification is the integration of reaction and separation within a single apparatus, achieving both thermodynamic and kinetic advantages. Reactive distillation (RD) exemplifies this integration, allowing continuous removal of water by-product, thereby shifting chemical equilibrium toward ester formation in line with Le Chatelier’s principle. Studies report energy savings of up to 80% compared to conventional batch processes [107]. Innovations like reactive dividing wall columns (RDWCs) further enhance performance by combining multistage separation and reaction zones within one unit, reducing capital costs and CO_2_ emissions. This allows savings from 11.6% to 58.65% [106,108,109].

Recent advancements have also yielded hybrid systems that integrate RD with pervaporation membranes. These hybrid processes capitalize on selective water removal and catalytic activity within the membrane structure, improving both conversion and selectivity, especially under equilibrium-limited conditions [110,111].

Another intensification strategy involves transitioning esterification reactions from liquid-phase systems to gas–liquid interfaces. Microbubble-mediated esterification leverages enhanced interfacial area to improve mass transfer and reaction kinetics. Compared to conventional processes, where methyl acetate formation typically reaches 78% conversion after 60 min, the microbubble approach achieves up to 96% conversion in only 20 min, using reactive distillation [112].

### 4.1. Thermokinetic Foundations and Reactor Design

Thermodynamically, esterification reactions are mildly endergonic and equilibrium-limited. As previously outlined, water removal shifts equilibrium and modifies kinetic behavior. In lipase-catalyzed systems, this also affects enzyme conformation (Section 3.1). A detailed discussion of membrane-based water removal is provided in Section 5 and Section 6. In lipase-catalyzed systems, water activity influences enzyme conformation and turnover rate [113].

In continuously operated reactor systems with integrated water removal-such as reactive distillation columns, the classical Michaelis–Menten kinetics are fundamentally altered. While the original model assumes constant substrate and product concentrations under reversible equilibrium conditions, selective water removal (e.g., via azeotropic distillation) renders the reaction effectively irreversible. Studies have shown that targeted water extraction effectively lowers the apparent Michaelis constant, as the reverse reaction is suppressed. Additionally, functionalized catalysts featuring hydrophobic pore architectures or specific affinity towards alcohol or water molecules can further shift the equilibrium by selectively adsorbing or repelling reaction components. This enhances the apparent turnover frequency and improves both catalytic efficiency and space-time yield [106].

The choice of an appropriate reactor type is largely influenced by thermodynamic constraints, mass transfer characteristics, and economic considerations. Comparative simulations reveal that continuously operated stirred tank reactors (CSTRs) can achieve slightly higher conversions (~2.3% more) than plug flow reactors (PFRs). However, this advantage comes at the cost of significantly higher operating temperatures (110 °C vs. 70–75 °C in PFRs). In contrast, PFRs offer longer operational lifetimes and improved thermal efficiency [114].

Table 8 summarizes key reactor types relevant for esterification, including their mass transfer behavior and kinetic advantages.

### 4.2. Microreactors and Hybrid Process Concepts

Microreactors have gained attention for their high surface-to-volume ratios, which enable excellent heat and mass transfer performance. Typical values: Sherwood numbers (3–4), Péclet numbers (>100), and Nusselt numbers (4–8) demonstrate these capabilities. Helical-flow microreactors further enhance conversion through intensified mixing and short residence times (~120 s for >95% yield) [120].

**Figure 2:**
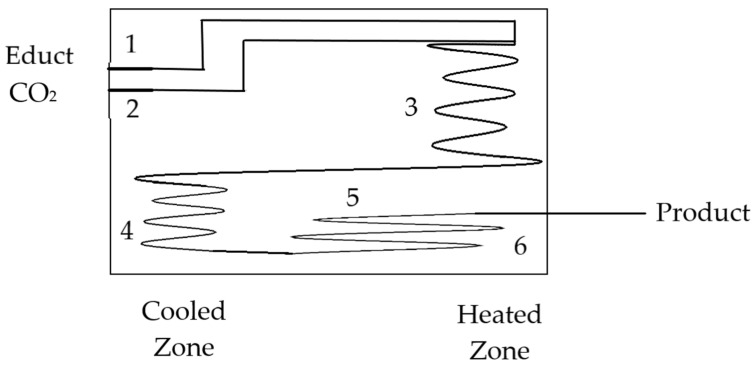
Schematic drawing of a microreactor for esterification under supercritical CO_2_ (90–110 bar). Phthalic anhydride and methanol enter at (1), CO_2_ at (2). The reaction occurs in zone (3). Flow and pressure are regulated by (4). After expansion in (5), the product exits at (6). The reactor includes a cooled and a heated zone [121].

Figure 2 shows a schematic of a continuous-flow microreactor designed for the esterification of phthalic anhydride with methanol under high-pressure and/or supercritical CO_2_ conditions (90 and 110 bar). These CO_2_ conditions are highly energy-intensive and challenging to implement in large-scale industrial processes. The system includes components: phthalic anhydride and methanol are introduced through inlet (1), while liquid CO_2_ is fed through inlet (2). The reactants enter the reaction zone (3), where esterification occurs under controlled temperature and pressure. A fluidic resistor (4) regulates flow and pressure within the system. After the reaction, the mixture passes into the expansion zone (5), allowing pressure reduction before the product exits via outlet (6). This microreactor setup enables efficient esterification by combining continuous processing with supercritical CO_2_ conditions. The supercritical CO_2_ conditions are described in more detail in Section 6.2 [121].

Catalytic coatings, such as ion-exchange resins or immobilized lipases, enable reaction within seconds to minutes. Static and dynamic mixing devices, including Corning Advanced-Flow™ and Nagoya Institute of Technology (NiTech) oscillating reactors, have been benchmarked for enzymatic esterification [122]. Hybrid processes incorporating RD and membrane modules provide synergistic benefits. Simulation studies [32,123] indicate that combining pre-reactors, side-reactors, and extractive zones can optimize conversion and separation. Advanced process control methods, including model predictive control and reinforcement learning, are increasingly applied [106].

Overall, process intensification in esterification represents a multifaceted strategy combining reactor design, thermokinetic insights, catalytic enhancements, and digital control concepts. This convergence paves the way for highly efficient, scalable, and sustainable ester production while offering flexibility for integration of novel catalytic systems and separation technologies.

## 5. Membrane Systems in Esterification

Membrane systems have increasingly attracted attention in esterification processes due to their potential to combine reaction and separation. Pervaporation membranes and catalytic membrane reactors offer a significant advantage by enabling continuous water removal during the reaction, thereby shifting the equilibrium toward ester formation and enhancing conversion beyond equilibrium-limited yields [124].

Among available membrane technologies, zeolite-coated ceramic pervaporation membranes have shown promising results. These systems feature a selective zeolite layer that enables preferential water permeation while also providing catalytic activity. Catalysis and separation can be integrated into a single layer or arranged in separate functional layers, enabling independent optimization of reactivity and selectivity. Large-pore zeolites, such as Y-type structures, have demonstrated notable catalytic performance in esterification reactions like the conversion of acetic acid with butanol, offering both catalytic sites and molecular sieving functionality [125].

Recent studies confirm that continuous pervaporation membrane reactors outperform traditional batch and recycle-loop configurations. Continuous operation ensures a stable driving force for water removal, maintaining high conversion, and supporting a compact process design. Localized water removal at the reaction interface suppresses hydrolysis and enhances ester accumulation, yielding improved performance compared to inert pervaporation systems [124,125].

In addition to zeolite-based systems, advances in membrane materials such as metal-organic frameworks (MOFs), covalent-organic frameworks (COFs), and mixed-matrix membranes (MMMs) expand the possibilities of membrane-assisted esterification. These materials offer tunable pore structures, reactive functional groups, and enhanced stability under demanding chemical conditions [126]. Hybrid membrane systems combining separation and product recovery—e.g., through nanofiltration or supercritical CO_2_ extraction-provide further opportunities for process intensification [127,128].

Selecting an appropriate membrane system for esterification requires consideration of the separation type (e.g., water removal, product enrichment), stability, membrane–reactant interactions, and catalytic compatibility. Pervaporation is ideal for continuous water removal, while nanofiltration suits selective product recovery or catalyst retention. Bifunctional membranes combine reaction and separation but involve more complex design.

Table 9 compares three membrane systems, pervaporation, nanofiltration, and bifunctional membranes, with respect to their application in esterification processes. Each system offers different strengths: pervaporation is well suited for continuous water removal, nanofiltration for product separation and catalyst retention, and bifunctional membranes for integrating reaction and separation. Key differences lie in their mechanisms, material requirements, thermal and long-term stability, and fabrication complexity. The choice of system depends on process demands such as selectivity, stability, and integration level.

### 5.1. Comparison of Different Membrane Systems for Esterification

Polymeric and ceramic membranes represent the two principal classes used in esterification processes. Polymer-based membranes such as polyvinyl acetate (PVA), polyetherimide (PEI), or polydimethylsiloxane (PDMS) are known for their high selectivity and ease of processing. Hydrophilic groups—such as hydroxyl or ether moieties—facilitate water uptake. The free volume fraction within the polymer matrix plays a key role in water permeability, with highly cross-linked structures showing better selectivity but reduced flux [133,134,135].

Ceramic membranes, particularly those coated with zeolites, exhibit significantly greater stability and resistance to thermal and chemical stress. Their porous structure and defined molecular sieving (0.3–0.5 nm) promote selective water removal. Zeolite types such as Linde Type A (LTA) or Zeolite Socony Mobil—five (MFI) offer high affinity to water molecules and can be optimized further through metallic doping (e.g., with Na^+^, K^+^, or Mg^2+^) [110,136].

Mixed-matrix membranes (MMMs) combine polymer-based support materials with inorganic nanofillers (e.g., silica particles, zeolites, or graphene oxide) and unite the advantages of both classes. They often exhibit improved permeability while maintaining or even enhancing selectivity. A critical aspect, however, is the uniform dispersion of the fillers, as agglomerates may lead to membrane defects [127,137].

**Figure 3:**
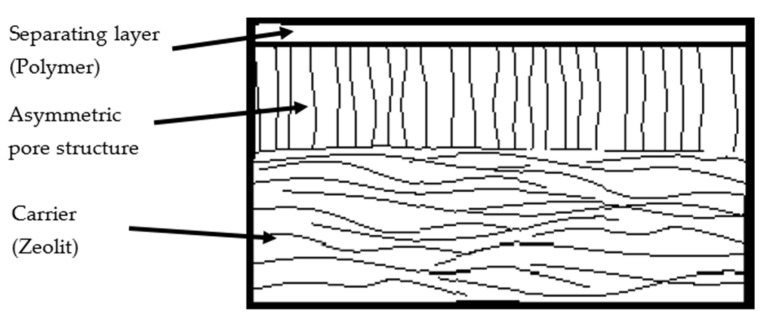
Cross-sectional schematic of a typical composite membrane, consisting of a thin selective separating layer and a porous support structure [128].

Nano-structured membranes, such as those based on MOFs or COFs, offer a highly tunable pore architecture. They provide increased functional diversity and can be precisely adjusted to molecular size and interactions with the reaction medium [138]. Supercritical CO_2_ membrane processes complement these options [139]. Figure 3 shows a schematic cross-section of a typical composite membrane. The separating layer, located at the top, is produced from polymeric materials and provides the membrane’s selective transport properties. It features a dense, vertically oriented morphology that supports high selectivity [128].

This intermediate region facilitates efficient mass transport. At the base, a robust carrier-commonly composed of inorganic materials such as zeolites or other ceramics-ensures mechanical integrity, thermal resistance, and chemical stability under process conditions. The membrane architecture is characterized by the following layer dimensions, each contributing to selective transport and mechanical stability.

Separating layer: 0.5–2 µm
Asymmetric porous structure: 70–100 µm
Carrier layer: up to 100 µm

Transport of small molecules such as water typically proceeds via the sorption-diffusion-desorption mechanism: molecules are first absorbed at the membrane surface, then migrate through the membrane matrix driven by a concentration gradient, and are finally desorbed on the permeate side [128]. An overview of the properties of various membrane systems for esterification is provided in Table 10.

The compatibility between membrane materials and catalysts particularly in hybrid systems is a critical yet often overlooked factor. Strong acids or organic solvents used in esterification may degrade polymeric membranes (e.g., PVA, PEI), while enzymatic systems can be deactivated by polar solvents, elevated temperatures, or pH shifts. To ensure stable performance, careful material selection is essential. For example, ceramic and zeolite-based membranes offer superior chemical resistance, while enzyme–membrane hybrids require hydrophobic, low-water-activity environments to preserve enzyme activity. These aspects must be considered in the design and scale-up of hybrid reactors

### 5.2. Alternative Membrane Separation Processes for Esterification

In addition to pervaporation, other membrane separation processes are gaining increasing relevance. Nanofiltration, electrodialysis, and supercritical CO_2_ membrane processes each offer specific advantages. Nanofiltration is particularly well-suited for the selective separation of low-molecular-weight esterification products. It operates based on steric exclusion and electrochemical interactions. Donnan-exclusion mechanisms play a significant role. Negatively charged membrane surfaces, enhance the retention of anions while allowing selective passage of cations, which typically introduced via sulfonic acid groups [20,142,144].

Electrodialysis offers the potential to separate polar and nonpolar components, particularly in reactive systems containing charged intermediates or in processes that combine esterification with neutralization or ion exchange steps [145]. However, this technique requires precise control of process parameters, such as pH and ionic strength. Supercritical CO_2_ membrane separation leverages the excellent solvent properties of CO_2_ in its supercritical state. This is explained in Section 4.2 and Section 6.2 [73,146,147].

### 5.3. Molecular and Technical Aspects of Pervaporation in Biocatalytic Esterification

Acidic catalysts and polar organic solvents, such as methanol or short-chain carboxylic acids can induce swelling, chain scission, or hydrolysis in polymer-based membranes, particularly those containing ester, ether, or amide linkages. Enzymes used in hybrid systems are similarly sensitive: their catalytic activity may deteriorate in hydrophilic environments due to excessive water absorption, in response to pH fluctuations, or under elevated temperatures exceeding their thermal stability range [148,149].

To mitigate such degradation effects, ceramic and zeolite-based membranes are frequently employed due to their chemical inertness, thermal robustness, and resistance to acidic or oxidative media. Alternatively, polymeric membranes can be hydrophobically modified or crosslinked to improve solvent resistance and prevent leaching [149].

Pervaporation is a solution-diffusion-based membrane process, where hydrophilic polymers such as PVA, polyelectrolyte complexes, or sulfonated membranes selectively remove water from organic reaction media. Vacuum or inert gas sweeping on the permeate side maintains the driving force [91,150].

Recent innovations have led to the development of enzyme-integrated membranes, or hybrid biocatalytic membranes, in which the lipase is covalently bound to or entrapped within the membrane matrix. This approach allows simultaneous catalysis and separation within a single unit, eliminating external reactor-separator coupling and reducing pressure losses and dead volume [88,125].

### 5.4. Mechanistic Insights and Future Perspectives in Pervaporation-Assisted Esterification

Pervaporation has established itself as a central technology for the continuous removal of water during the esterification processes. It operates via the sorption-diffusion-desorption mechanism [2,147]. Membranes composed of hydrophilic materials such as polyvinyl alcohol (PVA), sulfonated polymers, or zeolite-coated ceramics are particularly effective due to their strong hydrogen bonding capacity [127,143].

In industrial-scale operations, pervaporation provides enhanced product yields by suppressing the reverse hydrolysis reaction and enabling higher steady-state conversions. This is especially beneficial in integrated setups, where reaction and separation are co-occurring. These systems frequently utilize catalytically active membranes composed of zeolites or MOFs, allowing for localized catalysis and water removal [124,151].

A noteworthy innovation is the development of hybrid biocatalytic membranes, in which enzymes such as lipases are immobilized or covalently bound within the membrane structure. These systems facilitate for simultaneous catalysis and separation, reducing dead volume and eliminating the need for external reactor–separator coupling. For instance, a tubular pervaporation module using Lipozyme thermomyces lanuginosa (TL IM) and a PVA membrane achieved full conversion in under 30 min at 50 °C, with high enzyme stability and reduced product inhibition [88].

MOF-based membranes, for example, function as both selective barriers and acid catalysts, while COF membranes offer precise control over pore size and functional group integration for advanced molecular discrimination [134].

Future advancements include smart membranes that respond to environmental stimuli such as temperature, pH, or voltage, enabling real-time control of permeability and selectivity. Hierarchically structured, multistage membrane systems are also under development, allowing simultaneous separation, catalytic reaction, and recycling within one compact and energy-efficient unit [126,132].

Taken together, these innovations are expected to position hybrid catalytic membranes as key enablers of sustainable esterification at industrial scale. Their ability to combine high thermal stability, tunable selectivity, and catalytic activity in a single platform supports solvent-free, energy-efficient, and low-waste processing-marking a significant step toward greener chemical manufacturing.

## 6. Emerging Technologies for Sustainable Esterification

This chapter explores three advanced strategies aimed at fostering sustainable esterification processes: mechanochemical esterification, the use of supercritical carbon dioxide (scCO_2_) as a reactive solvent, and microbubble technology for enhanced mass transfer. Despite their differing mechanisms, all three approaches share the common objective of overcoming classical limitations by leveraging novel physico-chemical principles.

Mechanochemical esterification relies on mechanical energy to activate reactants, enabling solvent-free transformations in the solid state. Supercritical CO_2_, owing to its non-toxic, inert, and readily removable nature, explained in Section 4.2 and Section 6.2. Microbubble systems, characterized by their exceptionally high specific surface area, drastically enhance gas–liquid mass transfer and facilitate rapid conversions [152].

Table 11 provides a comparative overview of selected experimental systems. It summarizes key parameters such as substrates, catalyst types, reactor configurations, and reported product yields [153].

In the following sections, these methodologies are examined in detail with regard to their chemical principles, reactor design, process intensification potential, and industrial scalability. Particular emphasis is placed on comparing these alternative technologies to conventional liquid-phase esterification, with the overarching aim of highlighting their capabilities to enable resource-efficient, selective, and scalable synthesis pathways.

### 6.1. Mechanochemical Esterification: A Solvent-Free and Green Alternative

Mechanochemical esterification is a model approach within the framework of green chemistry. It enables efficient synthesis under solvent-free conditions. Instead of relying on thermal activation, the reactants are driven to react via mechanical energy input: The rolling and collision of grinding media generate “hot spots”—localized zones of elevated temperature and pressure to facilitate bond activation and accelerate the reaction process [153].

This method has proven particularly effective for the synthesis of low-molecular-weight esters, such as those formed through the direct esterification of carboxylic acids with alcohols or the transesterification of fatty acid derivatives. For instance, long-chain fatty acids have been successfully converted in the presence of solid acid catalysts like Amberlyst-15 (explained in Section 2.2.1), achieving yields above 90% in less than 30 min. Mechanochemical conditions are also suitable for polymer-analogous reactions, including the formation of polyesters from diacids and diols, with high selectivity under solid-state conditions [154,159]. A major advantage of mechanochemical approaches lies in the complete elimination of solvents [160].

From a kinetic perspective, many mechanochemical esterifications follow pseudo-first-order behavior. Mechanical activation introduces lattice defects in the solid reactants, which increases their surface reactivity and enhances the adsorption of the reaction partners. Continuous abrasion and surface renewal of the catalyst particles under milling conditions further distinguish mechanochemistry from conventional homogeneous catalysis. This effect is particularly beneficial for the long-term stability of solid acid catalysts [154,158].

Reaction parameters such as milling ball diameter, filling degree, rotational speed, and milling vessel material (e.g., zirconia, stainless steel, or tungsten carbide) allow precise control over the system’s energy density, an essential factor for ensuring reproducibility and scalability. Notably, initial demonstrations using twin-screw extrusion have shown that these solid-state esterification processes can be successfully translated into continuous, industrial-scale operations [161].

Beyond classical carboxylic acid esterifications, mechanochemical techniques have been extended to more complex transformations. For example, enzymatic esterifications have been performed under mechanical activation conditions using immobilized lipases (Section 2.3). Furthermore, certain asymmetric transformations employing chiral catalysts have also been demonstrated under mechanochemical regimes [162,163].

Typical product yields in mechanochemical esterification range from 46% to 95%, with reaction times frequently below 30 min. In polymer chemistry, such solvent-free methods have proven effective for the production of polyesters and polyamides, including the high-yield synthesis of polylactic acid via direct polycondensation. This opens promising avenues for the development of sustainable materials [164,165].

### 6.2. Supercritical CO_2_ as a Reaction Medium for Sustainable Esterification

Supercritical carbon dioxide (scCO_2_) is an environmentally benign reaction medium due to its non-toxic, non-flammable, readily removable, and cost-effective nature. In lipase-catalyzed syntheses such as the production of geranyl acetate, initial conversions under scCO_2_ using biocatalysts like Lipozyme^®^ RM IM or Novozym^®^ 435 were relatively low (~12% after 4 h). However, substantial improvements, up to 60.5% conversion, were achieved through process optimization. These included adjusting the pressure to 120 bar, optimizing the CO_2_ flow rate (1.0 mL/min), fine-tuning the molar ratio of geraniol to acetic acid to 1:2, controlling the water content (2% *w*/*w*), and increasing enzyme loading (20 mg/mL). The optimal temperature was identified as 50 °C [166].

One of the most compelling aspects of scCO_2_ is its impact on the enzyme microenvironment. The unique physicochemical properties of CO_2_—particularly its ability to extract water from the enzyme’s active site into the gas phase—contribute to enhanced enzyme stability and sustained catalytic activity. In packed-bed reactors, conversions as high as 86% have been reported [166].

The supercritical state of CO_2_ exhibits gas-like viscosity combined with liquid-like density, resulting in excellent mixing behavior and enhanced mass transfer capabilities. Reaction parameters such as pressure (8–16 MPa) and temperature (45–65 °C) can be precisely tuned to optimize substrate solubility, enzyme conformation, and reaction kinetics [166]. In practical applications, both stirred tank reactors (STRs) and variable-volume reactors (VVRs) are employed. STRs allow for accurate control of mixing intensity, temperature, and pressure, while VVRs offer fine-tuning of system volume and pressure. Both reactor types rely on high-pressure CO_2_ pumps and precision pressure regulators to maintain steady-state operation.

The overall reaction kinetics are strongly influenced by the enzyme-to-substrate ratio, CO_2_ density, and the molar ratio of geraniol to acetic acid. The ecological advantages are particularly noteworthy: CO_2_ is readily available as an industrial byproduct, eliminates the need for volatile organic solvents, and reduces the overall energy input required for purification. Moreover, CO_2_ is not consumed during the reaction and can be continuously recycled within the process [166]. Nevertheless, maintaining enzyme activity under high-pressure conditions remains a substantial challenge. Improper pressure handling may lead to partial deactivation or conformational instability. This limitation has been successfully addressed through enzyme immobilization on solid supports such as crosslinked acrylic resins or ion exchange materials.

### 6.3. Microbubble-Assisted Esterification: Intensified Mass Transfer and Interfacial Reaction Control

Microbubble technology utilizes gas bubbles with diameters below 150 µm and exceptionally high specific surface areas. This significantly enhances gas-liquid mass transfer and provides a highly efficient interfacial environment for chemical reactions. In a representative experimental setup, methanol is vaporized and introduced into the liquid phase—typically oleic or acetic acid—through a porous borosilicate diffuser with a pore size of 90–150 µm, resulting in homogeneous microbubble formation. The reactor is maintained at 70 °C to prevent condensation and ensure bubble rise and stability. The excess of acetic acid facilitates immediate esterification at the bubble—liquid interface [158].

**Figure 4:**
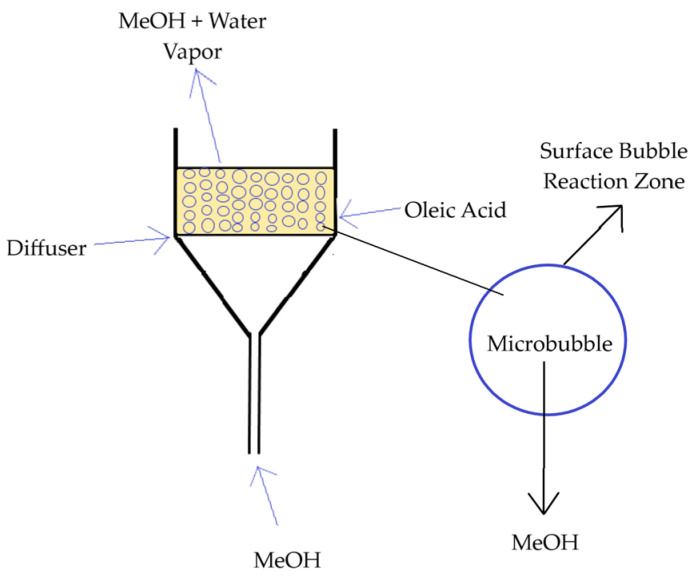
Schematic illustration of a microbubble reactor: methanol is introduced into oleic acid through a diffuser, forming microbubbles that create a reaction zone at their surface [5].

Figure 4 illustrates a schematic of the microbubble reactor configuration. Methanol is introduced at the reactor base and dispersed into the liquid phase via a porous diffuser, forming rising microbubbles. As the bubbles ascend through the liquid, a reaction zone is established at the gas—liquid interface [112].

A defining feature of this process is that the reaction occurs almost exclusively at the bubble surface, following a pseudo-first-order kinetic regime. The steep concentration gradient between the gas and liquid phases continuously drives the equilibrium toward ester formation. This effect is further amplified by the coalescence and bursting of bubbles at the liquid surface, generating enhancing mixing [156,158].

Careful selection of fitting diffuser materials is crucial for optimal reactor performance. In polar systems, such as acetic acid esterification, polar diffuser materials promote uniform bubble formation, while in non-polar media (e.g., biodiesel production), non-polar diffusers are preferred to prevent excessive bubble coalescence [167].

Response Surface Methodology (RSM) is employed in process optimization to evaluate the influence of key variables such as molar ratio, catalyst loading, premixing time, and liquid height. The resulting predictive model achieved high accuracy (R^2^ = 0.972). In parallel, a gated recurrent unit (GRU)-based artificial intelligence model was implemented, yielding a higher predictive performance (R^2^ = 0.998) [168].

Analytical monitoring was conducted using HPLC and gas chromatography to determine conversion and selectivity. Kinetic studies revealed that bubble diameter, gas flow rate, and reactor geometry are crucial determinants of process performance. Smaller bubbles reduce turbulent coalescence and maintain longer residence times, thereby improving conversion and mass transfer efficiency [169].

In addition to enhanced reaction kinetics, microbubble reactors offer notable energy savings. The high reaction rates can substantially reduce or even eliminate downstream purification steps, while enabling efficient catalyst utilization and reusability across multiple cycles. These factors reduce operational costs and improve overall process sustainability [156].

Future developments should aim to integrate microbubble reactors into continuous-flow and hybrid reactor systems, extending the benefits of enhanced interfacial mass transfer to other kinetically limited reactions, including transesterifications, hydrolyses, and enzymatic systems.

## 7. Industrial Implementation and Sustainability Economics

The industrial implementation of innovative esterification technologies requires a comprehensive assessment. While laboratory-scale research often emphasizes feasibility and selectivity, industrial-scale translation shifts the focus toward scalability, energy efficiency, sustainability, and overall cost-effectiveness [170].

The following three sections address key factors determining the industrial applicability of these technologies. Section 7.1 focuses on technological scale-up and highlights recent progress in reactor design and modularization strategies. Section 7.2 presents an in-depth sustainability analysis, evaluating energy consumption, carbon footprint, and resource utilization. Finally, Section 7.3 explores the economic framework and discusses the competitiveness of continuous esterification processes compared to traditional batch operations [118].

### 7.1. Technological Scale-Up of Membrane Reactors

Membrane reactors have become a key technology in industrial esterification processes due to their ability to efficiently combine reaction and separation. A particularly promising example is the continuous esterification of oleic acid with ethanol using niobic acid as a catalyst, which achieved a conversion of 71% at 249 °C and a molar ratio of 10.8:1 with a miniscule amount of catalyst. The system exhibited remarkable operational stability over several hours [171,172].

Pilot-scale implementations employing zeolite and ceramic membrane systems have demonstrated the feasibility of achieving internal membrane surface areas up to 20 m^2^ without significant selectivity losses (<5%). Such findings underscore the scalability potential of membrane-based reactor designs and highlight the role of membrane characteristics in optimizing conversion and selectivity [173].

The choice of suitable membrane materials plays a pivotal role in enabling effective catalytic performance and targeted separation. Polyethersulfonate (PES) membranes functionalized with sulfonated polystyrene grafts have shown notable promise. These membranes provide both catalytic sites and water adsorption capacity, thereby facilitating a shift in the thermodynamic equilibrium of esterification reactions. Under continuous flow conditions, internal membrane surface areas exceeding 25 m^2^ enabled conversion efficiencies of up to 93.8% within 10 s residence time at 30 °C and an ethanol-to-acetic acid molar ratio of 10:1 [173].

In parallel, membrane materials have been specifically developed to enable selective permeation during esterification, supporting process intensification efforts. Hydrophobic polydimethylsiloxane (PDMS) membranes are particularly effective for selective water removal, achieving permeation rates of up to 93%. Therefore, these membranes show formidable efficiency in promoting a reaction equilibrium shift toward ester formation. Conversely, hydrophilic polyvinyl alcohol (PVA) membranes favor alcohol permeation (up to 88%) and are better suited for systems in which retention of carboxylic acids is desirable while excess alcohol is selectively removed [118].

These transport properties enable process-specific membrane selection and integration into hybrid reaction-separation schemes. A particularly innovative approach involves enzymatic membrane bioreactors (MBRs). Herein, lipase-based systems have shown activity reductions of only 15% even after more than 3000 h of operation. Typical flow velocities in these systems range from 0.1 to 0.5 m/s, ensuring a balance between mass transfer and membrane integrity [142,144].

Catalytically active membranes such as Nafion™ tubes serve as catalysts and separation elements simultaneously, enabling higher selectivity while simplifying product isolation. These systems have increased the yield of n-butyl acetate from 70% to up to 95% via simultaneous water removal [174].

Furthermore, studies indicate that pervaporation membrane reactors (PVMRs) not only reduce energy consumption but also enable selective water removal at lower temperatures. These systems show a significantly improved CO_2_ footprint compared to conventional reactive distillation, especially when Zeolite catalysts like H-ZSM-5, H-MOR, or modified beta-zeolites are applied [130,175].

During scale-up, shear forces have to be managed carefully, as excessive mechanical stress can damage both the membrane and the catalyst. Simulation-based methods such as Response Surface Methodology (RSM) and Design of Experiments (DoE) are essential tools for identifying optimal operating conditions.

Pilot-scale membrane reactors optimized through such approaches have demonstrated up to 20% higher ester yields and up to 30% lower energy consumption [176]. Another representative case is the production of ethyl acetate using pervaporation systems for continuous water removal. A pilot-plant throughput of 500 L/h was achieved in one study, with selectivity losses remaining below 5%. Modular system design plays a decisive role in enabling further scale-up [177,178].

Membrane reactors are particularly attractive for industrial esterification because they inherently combine catalytic transformation with in-situ product separation, enabling continuous operation. A key advantage lies in the possibility of integrating heterogeneous catalysts directly onto or within the membrane surface, forming catalytically active membranes. This design minimizes mass transfer limitations, improves space-time yields, and reduces the need for catalyst recovery steps. Additionally, the coupling of catalytic and separation steps in close proximity enhances process intensification, leading to more compact reactor setups and reduced energy demands. These characteristics make membrane reactors a powerful and scalable solution for future-oriented chemical manufacturing, especially in applications where high selectivity and sustainability are essential [118,124].

The integration of reaction and separation within a single unit also demands finely tuned process conditions, which may not always be compatible across reaction and separation phases. In addition, the lack of standardized design guidelines for large-scale membrane reactor systems introduces uncertainty in scale-up and limits industrial confidence. Finally, challenges related to hydrodynamics, heat management, and sealing integrity further complicate the transition from lab-scale feasibility to full-scale application. These factors highlight the need for further research and development to tap into the full industrial potential of membrane reactor technology [179,180].

In conclusion, membrane reactors provide a highly efficient and flexible platform for continuous and sustainable esterification processes. They combine superior chemical performance with catalyst longevity and scalable adaptability for a range of industrial production scenarios. However, their industrial implementation remains limited due to several technical and economic constraints. High production and material costs, particularly for inorganic or composite membranes, represent a significant barrier to commercialization. Moreover, membrane stability under harsh reaction conditions is often insufficient, leading to degradation, fouling, and the need for frequent replacement, which increases maintenance requirements and operational downtime [181].

### 7.2. Sustainability Assessment of Emerging Technologies

The sustainability of modern esterification processes using membrane reactors is well documented and quantifiable. One of the most critical metrics is the significant reduction in CO_2_ emissions per kilogram of product. While conventional batch processes typically emit around 3.5 kg CO_2_ per kilogram of ester produced, membrane-assisted continuous processes achieve emission levels between 1.2 and 1.5 kg CO_2_ per kg of product. When powered by renewable energy sources such as photovoltaics, these emissions can be further reduced to below 1 kg CO_2_ per kilogram of ester [182].

A central driver of these improvements is the reduced energy demand. Enzymatic processes, particularly when combined with pervaporation, enable energy savings of up to 83% compared to traditional chemical esterification. This is largely due to the minimized thermal post-treatment, the prevention of reverse reactions through in situ water removal, and the low heat loss associated with continuous operation [118,183].

In such systems, material usage is also optimized. Membrane systems require up to 50% less catalyst per ton of product, offering both economic and environmental benefits. The extended lifetime of biogenic catalysts such as lipases—often produced from byproducts like oilcake—further reduces raw material consumption and supports social sustainability by fostering regional value creation [184,185].

Process design also significantly impacts waste generation. Transitioning from batch to continuous operation reduces wastewater volumes by up to 40% and solid waste by approximately 35%. In addition, modern pervaporation units can cut the cooling water demand by up to 53%, representing a crucial Environmental factor (E-factor) enhancement in the face of increasing global water scarcity [186].

Particularly sustainable process strategies combine renewable feedstocks (e.g., waste cooking oils), biogenic catalysts, energy-efficient separation techniques, and electricity from green sources. Together, these components form an almost closed-loop, low-impact system that aligns closely with global sustainability goals.

### 7.3. Economic Aspects of Continuous Esterification

The economic evaluation of continuous, membrane-based esterification processes reveals a clear trend toward superior profitability when compared to conventional batch operations. Key factors include capital and operating costs, feedstock quality, energy efficiency, process uptime, and regulatory incentives, all linked to sustainability [118].

Although membrane or enzymatic systems require higher initial investments, their significantly lower operating costs often result in a rapid return on investment (ROI). For instance, pervaporation-based systems often achieve a positive ROI within five years due to significantly reduced thermal energy requirements. Moreover, these systems eliminate the need for extensive distillation and minimize waste disposal costs [187].

Catalyst consumption is substantially lower thanks to extended service life, e.g., >3000 h for lipase-immobilized membranes, which directly reduces operational expenditures (OPEX) per unit of product. The use of higher-quality raw materials (e.g., anhydrous ethanol instead of technical-grade ethanol) can also be economically beneficial, as it improves conversion and limits side reactions. Documented raw material savings of up to 30% have been reported [89,188].

Digital process control, particularly through AI-driven optimization, can boost space-time yield by 10–15% while reducing energy consumption. The integration of heat exchangers and solvent recovery (e.g., ethanol recycling) contributes to further improvements in process efficiency [189].

Simulations suggest that production scales starting from 500 t/a benefit significantly from economies of scale, yielding total cost reductions of up to 25%. These benefits continue to increase with plant size, making the technology especially attractive for large-scale applications such as biodiesel and fine chemicals productions [9,190].

Sustainable processes also offer strategic market positioning. CO_2_ certificates, reduced environmental taxes and levies, access to subsidies, and the ability to market climate-friendly products at premium prices provides tangible financial advantages. Companies adopting certified green esterification technologies can differentiate themselves and mitigate long-term regulatory risks.

## 8. Limitations and Future Perspectives

Despite significant progress in the intensification of esterification processes, substantial challenges remain that hinder their widespread industrial implementation. Modern reactor concepts, such as membrane reactors, microstructured systems, and hybrid catalytic approaches, demonstrate compelling performance metrics at the laboratory scale. Notably, they offer high selectivity, strong energy efficiency, and improved product quality. However, the transition to industrial-scale applications reveals both technological and methodological limitations. This section provides a systematic overview of key limitations related to reactor design, process modeling, material robustness, and sustainability, while outlining future research directions.

### 8.1. Technological and Engineering Limitations

The scale-up of micro structured reactor systems remains one of the most significant technical challenges in the field of process intensification. While systems such as microreactors and membrane modules demonstrate excellent performance in laboratory settings, particularly in terms of reaction rate and mass transfer, several limitations emerge during translation to industrial applications. These include increased pressure drops, non-uniform temperature profiles, and membrane fouling, especially under prolonged exposure to reactive organic media. In large-scale setups, achieving homogeneous phase mixing and well-defined residence times becomes increasingly difficult [158,191,192].

Long-term membrane stability poses another critical limitation. Polymer-based materials such as polyvinyl alcohol (PVA) have displayed a loss of up to 35% of their permeability after only 1000 h of operation at moderate temperatures. Although ceramic alternatives offer higher thermal stability, they remain prone to cracking under mechanical stress in large-scale systems [193,194].

Heterogeneous catalysts face similar challenges, including activity losses caused by coke deposition and leaching of active sites. These issues often require energy-intensive regeneration cycles to restore catalyst performance. Modular reactor designs that support the replacement of catalyst units during operation offer potential to enhance plant availability. However, industrial environments demand materials with substantially greater mechanical strength and chemical resilience than those employed in lab-scale studies [195].

Multifunctional membranes that combine catalysis and separation within a single unit present an innovative strategy. Preliminary experiments with MOF-based systems have demonstrated stable operation over several hundred hours. Nonetheless, scalable implementation strategies for industrial applications are still lacking. Biocatalytic systems employing immobilized enzymes also show promising stability under laboratory conditions, yet tend to lose activity rapidly under real-world operating conditions especially in response to high shear forces or fluctuating substrate concentrations. These limitations highlight the need for membrane and enzyme carrier systems specifically engineered for long-term use and industrial-scale process stresses [196,197,198].

An additional bottleneck lies in the limited integration of reaction, separation, and recycling within fully continuous processing environments. While the hardware-level coupling of such steps is already employed in pilot plants, functional integration at the system level remains underdeveloped. In the absence of such holistic approaches, the full potential of process intensification remains underutilized [199,200].

### 8.2. Methodological Limitations and Data Gaps

The comparative evaluation of different process intensification strategies is significantly hindered by methodological inconsistencies across the literature. Experimental studies vary widely in terms of substrate selection, catalyst composition, solvent systems, and operating conditions, making direct performance comparisons difficult or even misleading [201,202,203].

A particularly critical deficiency lies in the limited availability and inconsistent implementation of robust life cycle assessments (LCA). The few existing LCA studies are predominantly based on laboratory or pilot-scale data, which are only partially transferable to industrial-scale conditions. Key environmental indicators such as CO_2_ emissions, water footprint, resource consumption, and toxicity are often incompletely recorded or derived from simplified assumptions. This lack of comprehensive data leads to blurred or inconclusive sustainability assessments and introduces regulatory uncertainty and investment risk in industrial contexts [204,205].

Moreover, politically driven regulations, persistently high energy and considerable material costs present challenges for industrial stakeholders. The implementation of novel, sustainable technologies often entails notable upfront investments. However, robust, predictive models capable of quantifying long-term economic and ecological benefits remain an unmet need for industrial implementation [204,206].

In addition, the mathematical modeling of novel reactor systems remains underdeveloped. Configurations such as microbubble reactors, pervaporation-integrated systems, and bifunctional membrane reactors are predominantly characterized by empirical data, with limited availability of validated kinetic or transport models. The inherent complexity of multiphase interactions, unsteady-state operation, and non-linear reaction environments challenges the applicability of conventional modeling approaches. In the absence of reliable simulations, the optimization of operational parameters, process intensification, and digital control remains largely empirical and lacks predictive power [198,207,208,209].

### 8.3. Material Stability and Feedstock Availability

A major bottleneck in the implementation of modern esterification technologies lies in the limited chemical and mechanical stability of the materials used, as well as the availability and sustainability of suitable feedstocks. This issue is particularly critical for functional materials such as ILs and zeolith catalyzed systems, which are prone to hydrolysis, chemical degradation, or mechanical embrittlement, especially in aqueous environments or under elevated temperatures. Their structural integrity can deteriorate due to water uptake, pH fluctuations, and repeated thermal cycling, limiting their reusability and long-term performance. Although strategies such as surface functionalization, hydrophobic coatings, the development of water-stable IL components, and embedding into stabilizing matrix systems (e.g., core-shell architectures or supported ionic liquid phases, SILPs) have shown initial promise, further investigation is required [210,211].

In parallel, enzymatic systems exhibit a high degree of sensitivity to process-relevant stressors. Biocatalysts, such as lipases, rapidly lose activity when exposed to supercritical CO_2_, organic solvents, or fluctuating pH and temperature profiles. The disruption of hydration shells, through mechanical shear stress and solvent-induced inhibition, often lead to irreversible denaturation of the enzyme structure. Immobilization on MOF-based carriers, hydrophobic supports, or stabilization through the addition of protective agents such as sugar alcohols or salts has demonstrated encouraging improvements. However, comprehensive data on their performance under dynamic operating conditions remain scarce, impeding a systematic evaluation of their suitability for continuous processes [166,212,213,214,215].

On the feedstock side, resource availability and sustainability concerns further complicate process development. The use of critical metals in catalyst formulations including lanthanides, scandium, or cobalt is increasingly constrained by geopolitical supply risks and regulatory frameworks, such as REACH. As a result, research efforts are shifting toward alternative systems based on more abundant elements such as aluminum, iron, or silicon. Despite their higher availability, these systems often exhibit lower catalytic activity, reduced stability, or diminished recyclability, thus requiring further optimization [216].

## 9. Conclusions

Esterification remains a pivotal reaction across diverse industrial sectors. Recent developments in catalysis, reactor design, and membrane technology have significantly advanced its efficiency and sustainability. This review has outlined how emerging heterogeneous and biocatalytic systems, in combination with membrane-assisted separation and continuous processing, are redefining the landscape of ester synthesis. However, limitations such as material stability, process integration, and scalability continue to challenge widespread industrial implementation.

Building on these limitations, future strategies should also focus on the convergence of smart materials, digital process control, and modular plant architectures. The next generation of esterification processes will be characterized by their adaptability, resilience, and intelligent design. Stimuli-responsive membranes capable of porosity or surface chemistry alteration in response to temperature or pH shifts will enable real-time separation optimization and fouling mitigation. 

Modular “plug-and-play” systems, combining reaction, separation, and analysis in compact, scalable units, offer additional flexibility. These architectures support product switching, maintenance, and system upgrades while enabling continuous quality control aligned with principles of new industry design At the material level, the integration of enzymatic and metal-based catalysis into hybrid catalysts, such as MOF-based supports, unlocks new opportunities for tandem reactions with enhanced selectivity and reduced process steps [217].

Altogether, these advances signal a paradigm shift: from conventional, energy-intensive batch operations toward integrated, intelligent, and resource-efficient esterification processes. By aligning technological innovation with ecological and economic imperatives, the chemical industry can establish esterification as a model for sustainable and digitally enabled production systems. The vision is clear an adaptive, modular, and high-performance process platform that spans from precise laboratory synthesis to smart, networked large-scale manufacturing. In addition to evaluating process efficiency, future studies should prioritize compatibility assessments between catalysts and membrane materials to ensure long-term stability in hybrid or continuous configurations.

This review addresses a key gap in the current literature by systematically integrating insights from catalysis, membrane separation, and reactor engineering. By aligning material selection, process design, and operational stability, we propose a framework for evaluating esterification processes under realistic industrial conditions.

## Figures and Tables

**Table 1:** Overview of various homogeneous acid catalysts for the esterification reaction.

Catalyst	Thermal Stability	Replicability Conditions	Application	Process Mode	Reaction Conditions	Yield (%)	Reference.
Hydrochloric acid (HCl)	Moderate *	Not measured *^3^	Acrylic acid with ethanol	Batch	50–70 °C, 7 h, atmospheric pressure	53.3	[10]
p-Toluenesulfonic acid (p-TSA)	Moderate	Not measured	Palmitic acid with deuterated ethanol	Batch	80 °C, 4 h, atmospheric pressure	53–99	[16]
Brønsted-acidic ionic liquids (ILa)	Very high *^2^	Different settings *^4^	n-caprylic acid with methanol [HSO_3_-pmim]^+^ HSO_4_^−^	Batch	90 °C, 3 h, 6 wt%; atmospheric pressure	96.2	[17]
Ionic liquid (1-(4-sulfonic acid) butylpyridinium hydrogen sulfate)	Very high	Different settings	Lauric acid with methanol IL (1-n-butyl-3-methylimidazolium bis(trifluoromethylsulfonyl)imide) Lauriv acid with methanol	Batch	70 °C, 5 h, atmospheric pressure	60	[18]
Phosphoric acid (H_3_PO_4_)	Moderate	Different cycles *^5^	Maize oil to ethyl and methyl esters induced by microwave irradiation	Monomode reactor	70 °C, 20 min, 3 wt%; atmospheric pressure	11	[19]
Sulfuric acid (H_2_SO_4_)	High *^1^	Different setting	Acrylic acid with ethanol	Batch	70 °C, 6 h, 3 wt%; atmospheric pressure	83.9	[20]
Methanesulfonic acid (MSA)	High	Different setting	Free fatty acids present in jatropha oil with methanol	Batch	60 °C, 4.5 h, 1 wt% atmospheric pressure	97.8	[21]
Dodecylbenzenesulfonic acid (DBSA)	High	Different setting	Lauricacid with 3-phenyl-1-propanol	Batch	40 °C, 170 h, atmospheric pressure	84	[22]
BF_3_	Moderate	Different setting	Formic acid and methanol	Batch	55–60 °C, 45 min, atmospheric pressure	95	[23]

* Moderate—Stable up to approximately 150 °C, lose activity under longer reaction times or aggressive conditions. *^1^ High—Stable up to 200 °C, without significant degradation. *^2^ Very high—Stable above 200 °C, even under harsh conditions such as long reaction times or near the boiling temperature of the solvent *^3^ Not measured—No information on repeatability, number of experiments, or standard deviations reported. *^4^ Different setting(s)—Data obtained under different experimental conditions, typically without statistical evaluation or indication of repeated trials. *^5^ Different cycles—Indicates experiments repeated in multiple cycles but without detailed data on reproducibility (e.g., variance, error bars).

**Table 2:** Overview of selected solid acid catalysts based on sulfonic acid functional groups for esterification reactions.

Catalyst	Thermal Stability	Functional Groups	Process Mode	Application	Yield (%)	Reference
Nafion NR50	Up to 125 °C	-SO_3_H	Batch	Hexanoic acid, ethanol	98	[46]
Amberlyst 70	Up to 190 °C	-SO_3_H	Batch (Autoclave)	Levulinic acid, 2-furylmethanol	80.2	[45]
Amberlyst 15	Very stable	-SO_3_H	Batch	Cellulose, acetic anhydride, acetic acid	44	[44]

**Table 3:** Summary of selected zeolite-based solid acid catalysts used in esterification and related acid-catalyzed processes.

Catalyst	Thermal Stability	Functional Groups	Process Mode	Application	Yield (%)	Reference
H^+^-zeolite β (150)	High (>300 °C)	Medium/strong Brønsted acid sites	Batch	Succinic acid, phenol	79	[54]
H^+^-zeolite Y (100)	Higher than H+-zeolite β	Shift from Lewis to Brønsted acid sites	Batch	Succinic acid, phenol	4	[54]
Al^3+^ Montmorillonite	Higher than H+-zeolite β	Increased Si/Al, enhanced hydrophobicity	Batch	Succinic acid, phenol	10	[54]

**Table 4:** Provides an overview of selected mesoporous polymer- and silica-based catalysts and their performance characteristics in esterification reactions.

Catalyst	Thermal Stability	Functional Groups	Process Mode	Application	Yield (%)	Reference
SBA-15 *	High (mesoporous polymer)	p-Toluenesulfonic acid (-SO_3_H)	Batch	Biodiesel production	71.9	[56]
MCM-48-20HPW *^1^	High (reusable up to four cycles)	Tungstophosphoric acid (TPA)	Batch (Autoclave)	Palmitic acid, cetyl alcohol	96.6	[58]
IL/Fe-SBA-15 *^2^	Moderate (organic ionic liquid)	Brønsted + Lewis acid sites	Batch	Oleic acid, methanol	81.4	[61]
SBA-15-PrSO_3_H *^3^	High	Sulfonic acid-functionalized	Batch (Home designed reactor)	Biodiesel production from canola oil	78	[62]
FSM-16-SO_3_H *^4^	High (reusable ≥3 cycles)	Brønsted + Lewis acid, -SO_3_H groups	Batch	Benzaldehyd, dimedone, ethyl cyanoacetat	Up to 93 (polyhydroquinoline synthesis)	[63]
CMI-10 (MAS-C(A)) *^5^	Very high (steam + boiling water)	Tetrahedral Al, Si-O-Al framework groups	Batch (Autoclave)	General acid catalysis/potential for esterification	Not specified	[64]

* SBA-15—Santa Barbara Amorphous-15. *^1^ MCM-48-20HPW—Mobil Composition of Matter (MCM-48), Tungstophosphoric acid (HPW) loading (20 wt%). *^2^ IL/Fe-SBA-15—Ionic Liquid (IL), Santa Barbara Amorphous-15 (SBA-15) with Iron(III) nitrate nonahydrate. *^3^ SBA-15-PrSO_3_H—Santa Barbara Amorphous-15 (SBA-15), Pluronic suspended in 1M H_2_SO_4_ solution. *^4^ FSM-16-SO_3_H—Folded sheet silicate mesoporous material (FSM-16) functionalization of silanol groups on the. surface of FSM-16 with -SO_3_H groups. *^5^ CMI-10 (MAS-C(A))—Mesoporous aluminosilicates synthesized by Laboratoire de. Chimie des Matériaux Inorganiques (CMI) in acetone.

**Table 5:** Metal oxide-based catalysts for esterification of low-molecular-weight carboxylic acids and bio-oil components.

Catalyst	Structure and Modification	Acid Site Type	Process Mode	Substrate(s)	Yield (%)	Ref.
SO_4_^2−^/ZrO_2_-TiO_2_	Deposition-precipitation, 50 wt% ZrO_2_	Strong Brønsted acid	Batch	Recycled bio-oil, Acetic acid, ethyl acetate	18.4	[65]
PW-TiO_2_ (MOF-derived)	MOF-125(Ti) + H_3_PW_12_O_40_, calcined at 350 °C	Heteropolyacid, Brønsted acid	Batch (Autoclave)	Oleic acid (small acids also compatible)	90.5, stable over 6 cycles	[68]
80%CaO/Al_2_O_3_	co-precipitation	Brønsted + Lewis	Batch	Waste vegetable oil, methanol	98	[66]
ZrO_2_/SO_4_	Sol-gel, varied sulfate precursors	Brønsted + Lewis (precursor-dependent)	Batch	Oleic acid, methanol	96	[70]

**Table 6:** MOF-based catalysts for esterification and oxidation reactions.

Catalyst	Structure & Modification	Acid Site Type	Process Mode	Substrate(s)	Yield (%)	Ref.
S/MOF-101	One-pot synthesis with sulfonated linker	Brønsted (-SO_3_H)	Batch	Monocarboxylic acids, n-Hexanol	99	[73]
MOF-101(Cr)-SO_3_H	Sulfonic ligand + HCl mineralizer	Strong Brønsted (-SO_3_H)	Batch	Oleic acid (FFA) to methyl oleate (biodiesel) with methanol.	63.9	[74]

**Table 7:** Presents selected examples of immobilized lipases, carrier materials, and typical reactor applications.

Lipase	Material	Process Mode	Key Features	Ref.
Candida rugosa	Hydrophobic superparamagnetic microspheres	Batch/continuous	Magnetically separable, >2× activity vs. free lipase	[86]
Lipozyme RM IM	Polymeric packed-bed matrix (unspecified)	Continuous microreactor	High space-time yield, transferable to various substrates	[87]
Lipozyme TL IM	Hydrophilic membrane	Micro-pervaporation system	Very short reaction time, high product concentration 95.3% conversion	[88]
Novozym 435	Modified acrylic resin	Batch/Continuous	Widely used, highly stable in organic media	[84]
Not specified	Chitosan, epoxy-silicate, mesoporous silica	Hybrid reactor systems	High stability, compatible with membrane-based processes	[76]

**Table 8:** Operating parameters and features of intensified esterification reactors.

Reactor Type	Key Features	Mass Transfer Characteristics	Kinetic Advantages	Ref.
CSTR	Uniform mixing; tolerant to feed fluctuations; simple operation and maintenance	Moderate; depends on agitation speed	Suitable for slow kinetics; equilibrium-limited	[115]
Tubular reactor	High space-time yield; plug-flow behavior; better scalability	Gradient formation along length; axial diffusion	Higher conversion; allows reaction zone control	[116]
Reactive distillation	Combines reaction and separation; continuous water removal; integrated column design	Enhanced at vapor-liquid interface	Shifts equilibrium; improves yield at lower catalyst loadings	[117]
Membrane reactor	Selective water removal; catalyst immobilization possible; low energy demand	Controlled by membrane type and fouling	Reduces inhibition by water; shifts equilibrium	[118]
Microreactor	High surface-to-volume ratio; excellent heat control; very short residence times	Very high; Sherwood >3, Péclet >100	Reaction-controlled kinetics; rapid quenching possible	[119]
Microbubble reactor	Fine bubble dispersion; high interfacial area; improved interphase transfer in gas–liquid	Extremely high interphase transfer	Increased local reactant concentration; accelerates rate	[112]

**Table 9:** Comparison of pervaporation, nanofiltration, and bifunctional membranes for esterification. Processes with their respective separation type, mechanisms, materials, stability, fabrication complexity, and catalytic activity.

Parameter	Pervaporation	Nanofiltration	Bifunctional Membrane	References
Separation type	Water removal	Product purification, catalyst retention	Reaction and separation in one step	[124,125,127,128]
Mechanism	Sorption–diffusion–desorption	Pressure-driven, Donnan exclusion	Combined reaction and selective permeation	[128,129]
Materials	PVA, zeolite-coated ceramics	Sulfonated polymers, hybrid materials	MOFs, COFs, zeolite-modified membranes	[124,126,130]
Thermal stability	Medium to high	Medium	High	[124,126,130]
Long-term stability	High	Medium to high	Variable	[131,132]
Fabrication complexity	Low to medium	Medium	High (multilayered, catalytically active)	[124,126,130]
Catalytic activity	No (but can be combined)	No	Yes (e.g., zeolites, MOFs with active sites)	[124,126,130]

**Table 10:** Comparison of Selected Membrane Systems for Esterification Processes.

Technology	Substrate/Catalyst	Thermal Stability *^1^	Long-Term Stability *^2^	Applications/Special Features	Ref.
PERVAP™ 1131	PVA + SiO_2_	Medium	High	Suitable for lactic acid esters, chemically resistant	[139,140]
Zeolite membrane (LTA/MFI)	Zeolite-coated ceramic	High	High	Molecular sieving, strong hydrophilicity	[124]
Donnan nanofiltration membrane	Sulfonated polymers	Medium	Medium	Anion retention, Donnan-driven separation	[129,141,142,143]
MOF/COF Hybrid	MOF-101(Cr), COF-MOF	Medium–high	Variable	Combined catalysis and separation, functionalizable	[132,141]
Supercritical CO_2_ membrane	PDMS, MOF-based	High	Medium	Extraction of non-polar esters, solvent-free operation	[126,133,143]

*^1^ Thermal stability: High: >150 °C; medium: 80–150 °C; low: <80 °C. *^2^ Long-term stability: High: <10% performance loss after ca. 100 h of operation; medium: 10–30% performance loss; low: >30% performance loss, mechanical degradation.

**Table 11:** Comparison of selected microbubble, supercritical CO_2_, and mechanochemical processes, along with their typical substrates, reactor types, and reported yields.

Technology	Substrate/Catalyst	Reactor Type	Yield (%)	Ref.
Mechanochemistry	Dihydroquinazolinone/p-TSA	Ball mill	67–76	[154]
Mechanochemistry	Anhydride ring opening/cinchona alkaloid	Ball mill	60	[152]
Mechanochemistry	Cyclohexanols/acetic anhydride	Batch/Ball mill	up to 90	[153]
CO_2_-esterification	Geraniol + AcOH/Novozym^®^ 435	Stirred Tank Reactor (STR), Variable Volume Reactor (VVR), Packed Bed Reactor (PBR)	60–86	[155]
Microbubble biodiesel	Waste cooking oil/7% Sr-ZrO_2_/70 °C/20 min	Bubble reactor	85	[156]
Semi-batch bubble reactor	Oleic acid with ethanol/catalyzed with an acid	Bubble reactor	95	[157]
Microbubble reactor	Methanol/acetic acid/para- toluene sulfonic acid	Bubble reactor	91	[158]

## Data Availability

Not applicable.
